# A method quantifying soil melanin to understand fungal impacts on soil carbon stability

**DOI:** 10.1038/s41467-026-78616-4

**Published:** 2026-10-11

**Authors:** Sylwia Adamczyk, Bartosz Adamczyk

**Affiliations:** Natural Resources Institute Finland (Luke), Helsinki, Finland

**Keywords:** Carbon cycle, Chemical ecology

## Abstract

Fungal necromass, particularly dead microbial cells rich in melanin, is an important contributor to long-term soil carbon stabilization. Progress in understanding the role of soil melanin is limited by the lack of validated analytical methods. DNA-based approaches estimate only the genetic potential for melanin production and therefore provide indirect evidence of melanin occurrence. To directly assess soil melanin, we evaluated spectroscopic and spectrophotometric assays; however, both approaches proved insufficiently specific due to interference from chemically related compounds. Here, we introduce a liquid chromatography method that enables quantification of major fungal melanin types in soils. By enabling direct quantification of fungal melanin in soil, this framework allows longstanding hypotheses regarding melanized fungal necromass and soil carbon stabilization to be tested mechanistically. Here, we show with the proposed method that soil melanin concentrations are strongly correlated with total soil carbon, supporting a central role for fungal melanin in long-term soil carbon stabilization.

## Abstract (web summary)

A method developed to directly measure fungal melanin in soil, reveals a strong link between melanin and soil carbon and providing evidence for its role in long-term carbon stabilization

## Introduction

Soil organic matter is the largest terrestrial carbon (C) reservoir^1,2^. Its formation and persistence are governed by microorganisms (fungi and bacteria), and their interactions with plants, soil and are further shaped by environmental conditions^3–5^. While microbes function as primary decomposers, they also contribute to soil C stabilization, as their biomass is transformed into microbial residues (necromass), which are stabilized in the soil through aggregation, mineral association, and chemical persistence^2,5^. Recent evidence highlights fungal necromass as a globally dominant contributor to stable soil organic C (SOC), particularly across high–latitude regions^6^. This dominance is increasingly attributed to the biochemical composition of fungal cell walls, especially melanin, a structurally complex and highly resistant polymer that markedly enhances the long–term preservation of fungal necromass in soil^6–9^. In colder climates, fungal communities tend to display darker pigmentation, suggesting a higher prevalence of melanized taxa in C–rich high–latitude ecosystems^10^. Fungal melanin composition varies among species^11^ and between phyla: basidiomycetes primarily produce nitrogen-containing eumelanin, rarely also pheomelanin, whereas ascomycetes synthesize nitrogen-free, 1,8-dihydroxynaphthalene (DHN)-derived allomelanin^12^. Interestingly, plants can also produce melanin-like polymers, though via distinct, non-DHN pathways^13^, suggesting convergent strategies for producing recalcitrant pigments in both fungi and plants. As recently proposed, melanized root–associated fungi form an ecological group that strongly influences ecosystem processes in plant–soil systems, and owing to their beneficial effects on plant and soil health, fungi that produce significant amounts of melanin could be used as potential bioinoculants^14^. However, the absence of methods to directly measure fungal melanin in soil has left their ecological role and potential use in soil management largely untestable.

The potential of fungi to produce specific types of melanin can be estimated via DNA analyses^11^. However, as the melanin production varies with environmental conditions and not only with fungal species^15^ DNA–based methods alone have limited utility. For example, in *Meliniomyces bicolor*, melanin content can shift more than threefold with changes in growth conditions^7^. In addition to DNA–based prediction of melanin content, there is also a spectrophotometric method that uses Azure A as a reagent binding to melanin; however, the effectiveness of this method has been demonstrated only for fungal cultures and fungal necromass incubated in the soil in mesh bags^16^. In addition, numerous methods to characterize eumelanin and pheomelanin have been developed for clinical applications, including spectroscopy (e.g., Fourier–transform infrared spectroscopy, FTIR)^17^ however, these methods have not been tested with soil samples. Thus, the available methods do not allow us to study the soil melanin quantity and quality, limiting our ability to evaluate the role of fungal melanin in soil C stabilization.

**Fig. 1:**
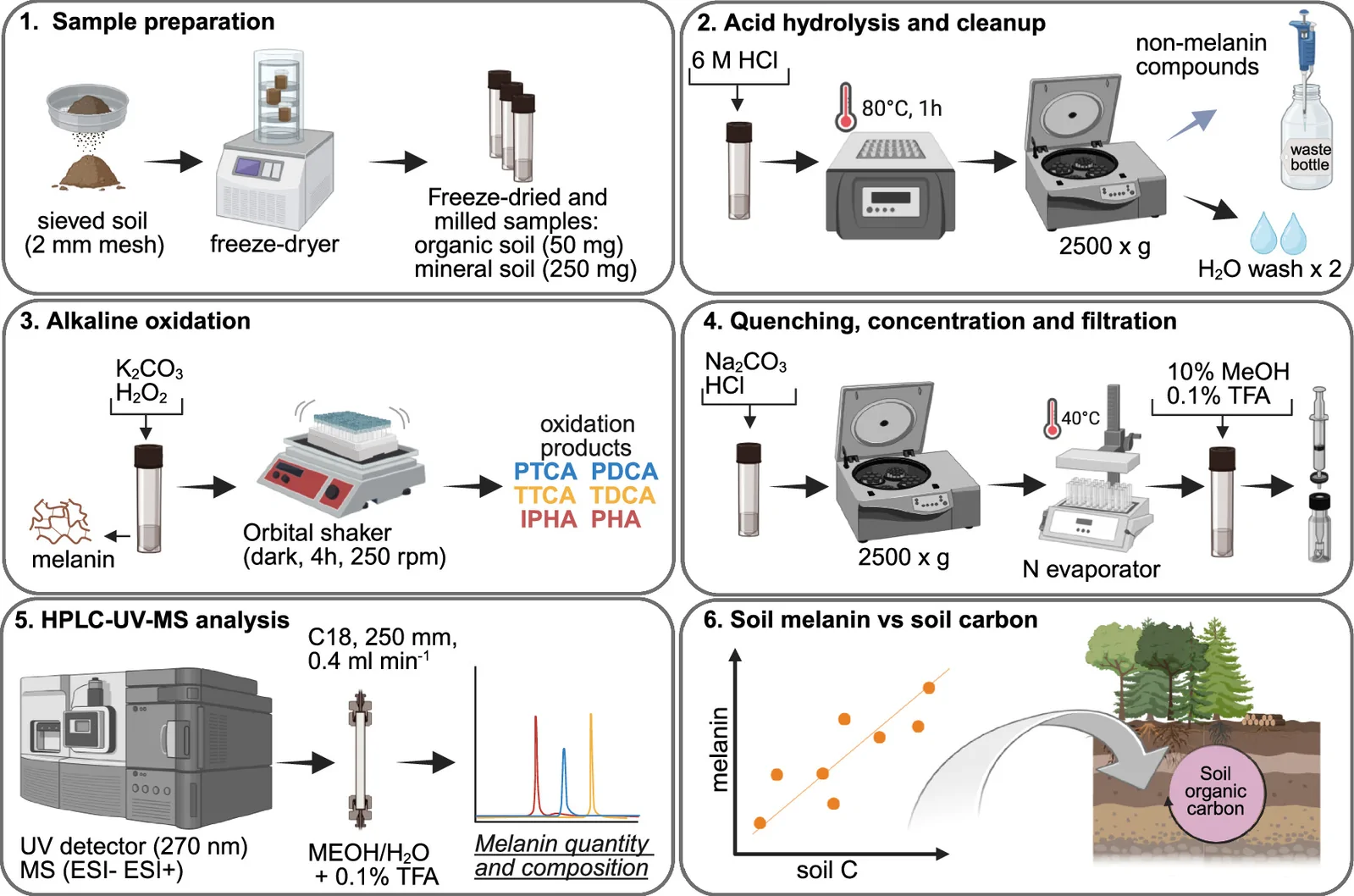
Diagram of the proposed method for measuring soil melanin. PTCA, PDCA, TTCA, TDCA, IPHA and PHA are alkaline oxidation products of pheomelanin, eumelanin and allomelanin, respectively. ACN acetonitrile, ESI electrospray ionization, ISM isocratic solvent manager, MS mass spectrometer, TFA trifluoroacetic acid. Created with BioRender.com.

In this work, we establish a framework for direct chemical quantification and differentiation of fungal melanin in soil. Beyond analytical development, this approach transforms fungal melanin from a trait inferred indirectly through DNA into a directly measurable component of soil organic matter. We adapt and optimize an high–performance liquid chromatography (HPLC) method originally developed for quantifying melanin in hair and melanomas for use with soil samples to analyse eumelanin and pheomelanin via alkaline oxidation and quantification of melanin–specific markers^17,18^. This enables direct testing of hypotheses linking melanized fungal necromass to long–term soil C stabilization. We further establish alkaline oxidation markers for allomelanin and demonstrate their specificity for fungal allomelanin (see Fig. 1 for a diagram of the method and the Methods section for details). Finally, we compare our approach with methods used for laboratory–cultivated fungi, namely the Azure A spectrophotometric assay and FTIR, both of which show substantial overlap with non–melanin compounds and a lack of specificity for soil fungal melanin. In doing so, we demonstrate that previous approaches lack the specificity required to resolve fungal melanin pools within chemically complex soil matrices. We validate the proposed HPLC protocol using a range of soil samples and observe a strong correlation between melanin concentration and soil C, N and fungal necromass content. Together, these results establish fungal melanin as a directly measurable soil biogeochemical pool and suggest that fungal melanization may represent a key mechanistic trait underlying persistent soil carbon stabilization.

## Results

### Validation of alkaline oxidation products

**Fig. 2:**
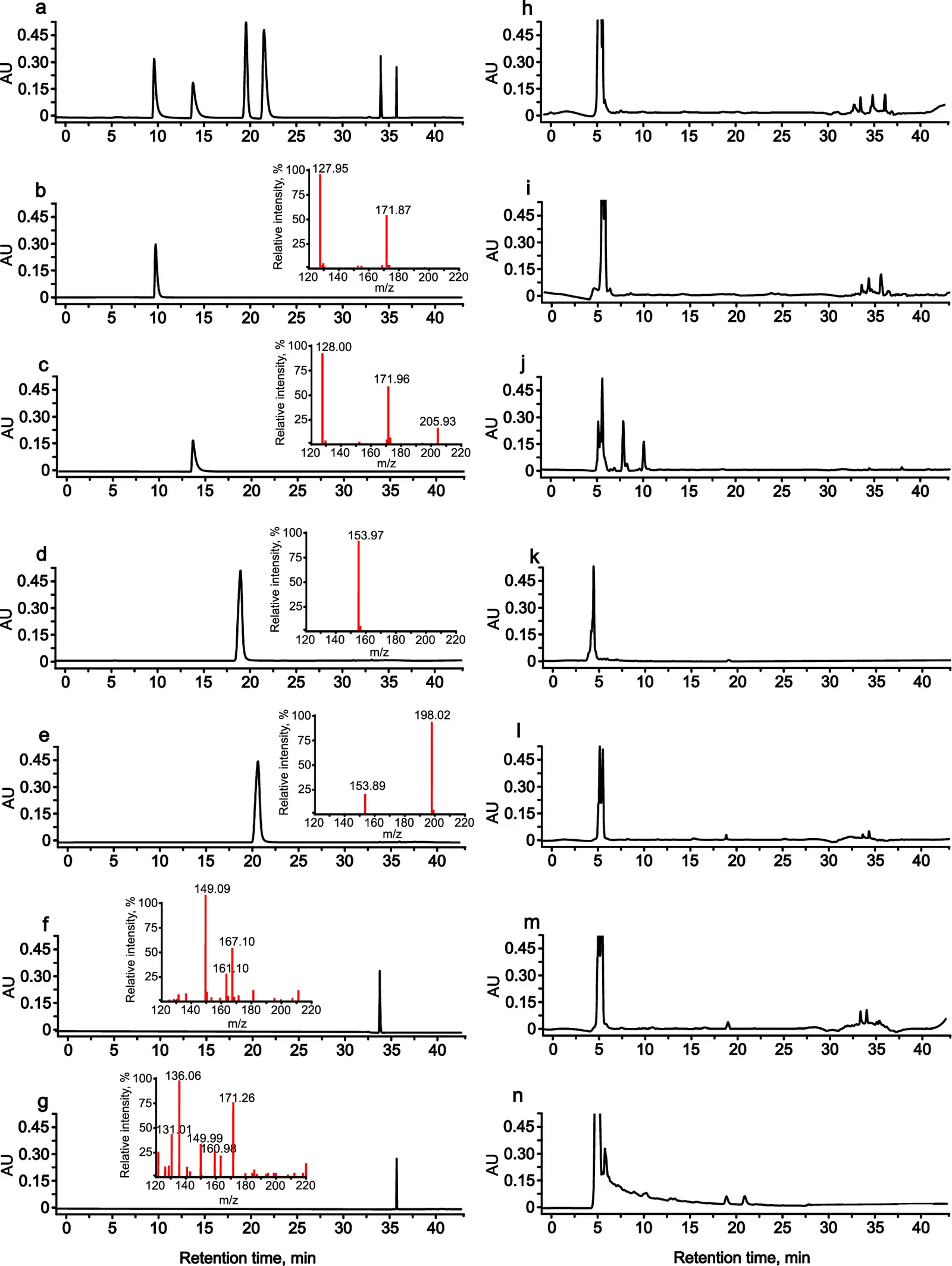
Chromatograms of products of melanin alkaline oxidation. **a** All standards together. **b** TDCA. **c** TTCA. **d** PDCA. **e** PTCA. **f** PHA. **g** IPHA. **h** synthetic allomelanin. **i** *Cenococcum*. **j** *Mortierella*. **k** *Tylospora*. **l** Forest mineral soil. **m** forested peatland. **n** synthetic eumelanin (see Supplementary Table 2 for soil characteristics). For TDCA, TTCA, PDCA, and PTCA mass scans are presented in ESI negative ion mode, and for PHA and IPHA mass scans are presented in ESI positive ion mode. For chemical structures of melanin and their products of alkaline oxidation see Suppl. Fig. 2.

Alkaline oxidation products used as standards for pheomelanin (thiazole–4,5–dicarboxylic acid, TDCA, and thiazole–2,4,5–tricarboxylic acid, TTCA) and eumelanin (pyrrole–2,3–dicarboxylic acid, PDCA, and pyrrole–2,3,5–tricarboxylic acid, PTCA) were very well separated under the proposed chromatographic conditions (see Fig. 2a). The peak purities were verified by mass spectrometer (MS) (Fig. 2b–e). For allomelanin, we identified two alkaline oxidation products, phthalic acid (PHA) and isophthalic acid (IPHA), as reliable markers (Fig. 2f, g). Although we evaluated a wide range of potential allomelanin markers, PHA and IPHA were the most suitable, as they consistently appeared in oxidation products of synthetic allomelanin (Fig. 2h), in fungi known to produce allomelanin (*Cenococcum*, Fig. 2i), and in soil samples (see Supplementary Table 1 and Supplementary Fig. 1 for other candidates of allomelanin markers). These markers of the three types of fungal melanin were further tested using fungal necromasses (Fig. 2i–k) and soil samples (Fig. 2l, m, see Supplementary Table 2 for soil characteristics) in relation to synthetic melanins (eumelanin and 1,8-DHN allomelanin, Fig. 2h, n). The peak purities for all the quantified compounds were confirmed with a MS with electrospray ionization (ESI) (see Fig. 2 for chromatograms and mass scans, Suppl. Fig. 2 for chemical structures and Suppl. Figs. 3–8 for key fragment ions of melanin markers).

### Alkaline oxidation vs. reductive hydrolysis

We also compared alkaline oxidation with alternative method, reductive hydrolysis providing instead of TTCA and TDCA (markers of pheomelanin) two other products: 4–amino–3–hydroxyphenylalanine (4–AHP) and 3–amino–4–hydroxyphenylalanine (AT). However, only standards were well separated and in soil samples the chromatograms were of low quality (see Suppl. Fig. 9 for chromatograms and description of reductive hydrolysis).

### Effect of HCl incubation on production of melanin markers

The HCl treatment applied to remove impurities from soil extracts may also affect the melanin polymer itself. Thus, we conducted in–depth studies of the effect of HCl incubation time and temperature, which proved that 1 h of HCl at 80 °C provides optimal conditions for preparation of the samples before alkaline oxidation (see Suppl. Tables 3 and 4). Short incubation with HCl (1 h) did not affect the structure of melanin; however, prolonged incubation altered the structure (see Suppl. Fig. 10 for FTIR results) and reduced the subsequent production of alkaline oxidation markers (Suppl. Table 3).

### Method specificity towards fungal vs. plant melanin

To evaluate the specificity of the method towards fungal allomelanins, we analysed a range of plant materials potentially containing plant melanin–like compounds, including seeds (pine and spruce), cones (pine and spruce), roots (pine and spruce), nut shells and black oat grains. In addition, we tested root–extracted tannins and commercial lignin as plant–derived compounds with potential to interfere with these analyses (see comparison with the Azure A method and FTIR, below). Alkaline oxidation of these plant materials did not yield fungal allomelanin markers (IPHA and PHA) (Suppl. Fig. 11). Trace amounts of allomelanin detected in roots were associated with the presence of fungi, as confirmed by the ergosterol measurements (marker of fungal biomass) shown in Suppl. Table 5.

### Azure A and FTIR methodology for detecting melanin

**Fig. 3:**
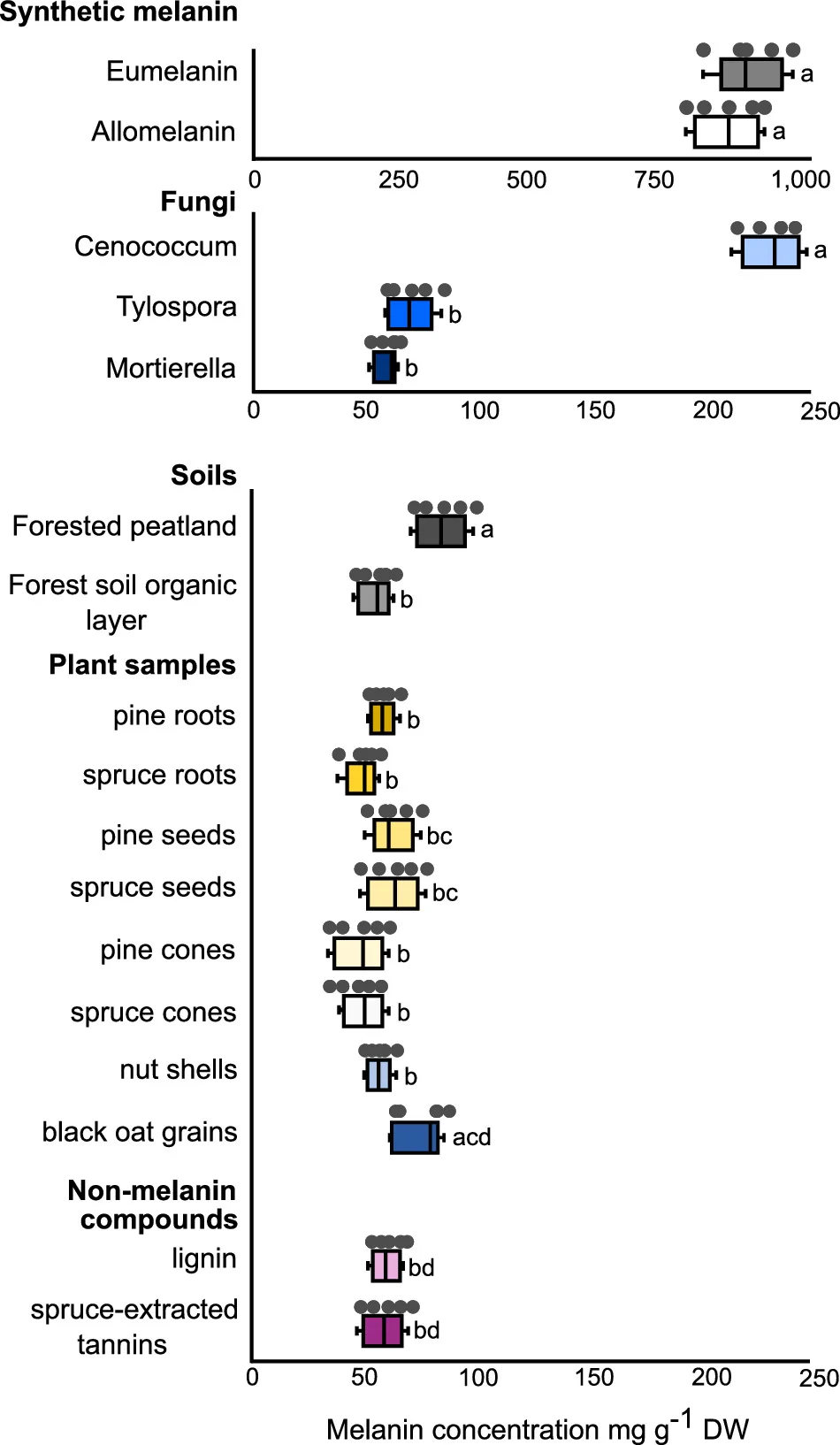
Concentration of melanin determined with the Azure A protocol. Significant differences are indicated by different letters (*p* < 0.05). Statistical comparisons were conducted separately for synthetic melanins and fungal samples, whereas soil, plant, and non–melanin compounds were analysed as a combined group. One–way analysis of variance followed by Tukey’s post hoc test was used. Box plots indicate median (middle line), 25th, 75th percentile (box) and 5th and 95th percentile (whiskers). *n* = 5. DW dry weight.

The same set of samples analysed with the proposed HPLC method was tested with spectrophotometric Azure A assay^19^ to enable direct comparison of the methods. The highest results were obtained for synthetic melanin and the fungal necromass of *Cenococcum* (Fig. 3). However, the assay also produced high apparent “melanin” values (> 30 mg g^−1^ DW) for several plant–derived materials—including pine seeds and cones—as well as for commercial lignin and root–extracted tannins, none of which contain fungal allomelanin (Fig. 3).

**Fig. 4:**
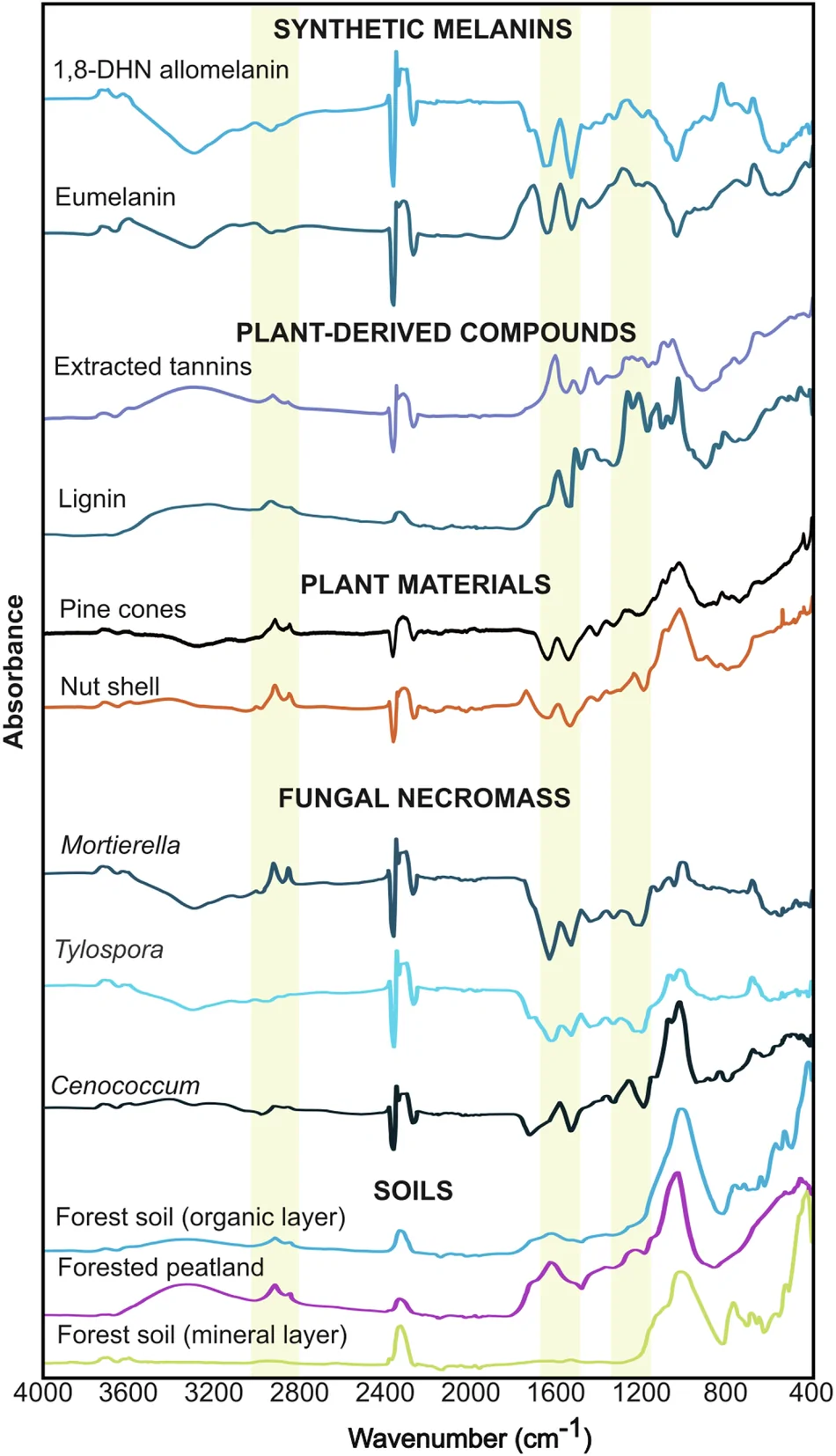
Examples of FTIR spectra of the samples. Marked in yellow column are the bands typical for melanin which overlap with the bands of polyphenolics.

We also compared the proposed HPLC protocol with FTIR using the same sample set. FTIR spectra revealed bands typically attributed to melanin: 3600–3000, 2927, 2858, 1650–1600 and 1300–1220 cm^−^^1^ assigned to –OH and –NH stretching, aliphatic C–H stretching, aromatic C = C stretching and/or conjugated C = O, and C–N/C–O stretching, respectively^20,21^. These FITR bands were detectable in synthetic melanin, fungal necromass and soil samples, but they overlapped with characteristic features of non–melanin samples such as lignin and spruce–extracted tannins (aromatic C = C stretching at 1650–1600 cm^−^^1^ and C–O stretching at 1265 cm^−^^1^). See Fig. 4 for the FTIR spectra.

### Limit of detection and quantitation

The linear range of the method was tested by multiple injections for each of the melanin oxidation products with a calibration curve at concentrations ranging from 0 to 0.5 mg mL^−1^. The limit of detection (LOD) and limit of quantitation (LOQ) were determined with the signal–to–noise ratio for each of the standards on the basis of the peak area with an ultraviolet detector (UV) (see Table 1). The LODs were defined at a 3:1 signal–to–noise ratio, and the LOQs were defined at 10:1 signal–to–noise.

**Table 1:** Limit of detection and quantitation, and linearity for HPLC with a UV detector

Marker	LOD (µg mL^−1^)	LOQ (µg mL^−1^)	linearity (R^2^)
TTCA	0.2	0.6	0.997
TDCA	0.2	0.6	0.997
PTCA	0.1	0.3	0.999
PDCA	0.1	0.3	0.997
PHA	0.5	1.5	0.995
IPHA	0.5	1.5	0.995

Alkaline oxidation in line with the proposed protocol for synthetic eumelanin, allomelanin and *Sepia* melanin resulted in linear PDCA/PTCA and PHA/IPHA production in the range of 0.1–5 mg. An alkaline oxidation time of 4 h was optimal, as a longer incubation time (up to 24 h) yielded even slightly lower results.

### Repeatability, precision, and percent recovery

The relative standard deviation (%RSD, often measured to describe repeatability, see Eq. 1) was calculated with the formula below and reached 1.4% for soil samples and 0.6% for standards (six replicates at the same HPLC run).

1 $$\%{{{\rm{RSD}}}}=\frac{{{{\rm{SD}}}}}{{mean}}x\,100\%$$

Intraday precision yielded the same results, with measurements taken in the morning, afternoon and evening (three replicates each). Interday precision, assessed using three samples measured across three consecutive days, resulted in %RSD values of 2.4% for soil samples and 0.75% for standards. We also estimated the percent recovery of standards after their addition to the soil (see Table 2, and Supplementary Table 6) and after interaction with representative soil clay minerals and iron oxides (kaolinite, illite, ferrihydrite, and goethite) (see Supplementary Table 7). Average percent recovery exceeded 80% for all soils and minerals.

**Table 2:** Percent recovery of markers of melanin and synthetic melanin added to soil (averaged for all used soils)

Marker/melanin	% recovery after addition to soil
TTCA	89(5.0)
TDCA	92(4.0)
PTCA	90(3.2)
PDCA	92(4.1)
PHA	80(6.2)
IPHA	79(7.7)
Synthetic eumelanin	82(8.8)
*Sepia* melanin	81(8.0)
Synthetic allomelanin	78(6.1)

In brackets standard deviation of three replicates. See Supplementary Table 6 for % recovery for each soil and Supplementary Table 7 for % recovery after reaction with soil minerals

### Total eumelanin and allomelanin calculations

We conducted alkaline oxidation of *Cenococcum* necromass (containing allomelanin), synthetic allomelanin, synthetic eumelanin and *Sepia* melanin (from 1 to 10 mg of each) using the proposed method. As *Sepia* melanin (commercial, 99% purity) and *Cenococcum* necromass (containing 20% melanin, as measured with Azure A in this study) provide more natural melanin signatures than synthetic melanins, as confirmed by the marker ratios (PTCA/PDCA and PHA/IPHA) that better reflect soil melanin characteristics (Table 3), we propose using natural melanin as the calibration standard. Accordingly, melanin concentrations should be calculated using these natural standards in accordance with Eqs. 2 and 3. In addition, percent recovery for studied soil should be taken into account.

2 $${{{\rm{Allomelanin\; concentration}}}}\left({{{\rm{mg\; g}}}}-1{{{\rm{soil\; DW}}}}\right) ={{{\rm{IPHA}}}}+{{{\rm{PHA}}}}({{\rm{mg}}}\; {{\rm{g}}} -1{{{\rm{soil\; DW}}}})\\ {xfAlx}(1/{Rf})$$

$${fAl}\,=M\,{ref}/\left({{{\rm{IPHA}}}}+{{{\rm{PHA}}}}\right){ref}$$

Where the percent recovery (*Rf*) is expressed as decimal fraction (i.e. 100% equal to 1), *f*Al – is a correction factor of alkaline oxidation products from allomelanin: M *ref* is known allomelanin mass of standard, and $\left({{{\rm{IPHA}}}}+{{{\rm{PHA}}}}\right){ref}$ are concentrations of IPHA and PHA from this known allomelanin standard.

3 $${{{\rm{Eumelanin\; concentration}}}}\left({{{\rm{mg\; g}}}}-1{{{\rm{soil\; DW}}}}\right)={{{\rm{PTCA}}}}+{{{\rm{PDCA}}}}\left({{{\rm{mg\; g}}}}-1{{{\rm{soil\; DW}}}}\right) \\ {xfEu\; x}(1/{Rf})$$

$${fEu}\,=M\,{ref}/\left({{{\rm{PTCA}}}}+{{{\rm{PDCA}}}}\right){ref}$$

Where the percent recovery (*Rf*) is expressed as decimal fraction (i.e. 100% equal to 1), *f* Eu is a correction factor of alkaline oxidation products from eumelanin standard (*Sepia* melanin): M *ref* is known eumelanin mass of standard, and $\left({{{\rm{PTCA}}}}+{{{\rm{PDCA}}}}\right){ref}$ are concentrations of PDCA and PTCA from this known eumelanin standard.

**Table 3:** Concentrations of oxidation products of melanin in various samples quantified with HPLC with a UV detector

Sample	Pheomelanin		Eumelanin		Allomelanin	
	TTCA	TDCA	PTCA	PDCA	PHA	IPHA
**Fungi**						
*Tylospora*	**-**	**-**	124(4.5)	22(2.3)	**-**	**-**
*Cenococcum*	**-**	**-**	**-**	**-**	184(15.1)	212(8.2)
*Mortierella*	90(2.9)	20(10.0)	**-**	**-**	**-**	**-**
**Melanins**						
Eumelanin	**-**	**-**	2600(9)	2400(11)	-	-
*Sepia* melanin			15001(11)	5012(14)		
Allomelanin	**-**	**-**	-	-	1402(10)	1599(21)
**Soil**						
Forest organic^A^	**-**	**-**	5.1(1.0)^a^	1.0(0.1)^b^	11.1(1.9)^b^	13.3(1.2)^ab^
Forest mineral^A^	**-**	**-**	1.2(0.2)^b^	-	3.1(1.2)^c^	-
Forest organic^B^	**-**	**-**	6.0(0.1)^a^	2.3(0.2)^a^	15.2(2.7)^b^	17.2(4.1)^a^
Forest mineral^B^	**-**	**-**	2.0(0.9)^bc^	-	2.1(0.1)^c^	-
Forest organic^C^	1.0(0.2)^a^		7.8(1.83)^a^	1.1(0.12)^b^	14.1(3.19)^b^	15.7(1.88)^a^
Forest mineral^C^	0.2(0.1)^b^	-	1.0(0.22)^b^	-	3.5(0.87)^c^	-
Forest organic ^D^	0.5(0.3)^b^		2.8(0.65)^c^	0.8(0.24)^b^	6.6(1.52)^b^	7.4(0.69)^b^
Forest mineral^D^	-	-	0.9(0.22)^b^	-	2.5(0.69)^c^	-
Forested peatland^E^	-	-	4.1(1.8)^ac^	0.9(0.2)^b^	31.3(3.1)^a^	9.4(3.7)^b^
Agricultural soil^F^	**-**	**-**	1.1(0.1)^b^	0.8(0.1)^b^	10.2(2.9)^b^	-
Agricultural soil^G^	-	-	0.8(0.12)^b^	0.6(0.09)^b^	7.2(1.01)^b^	-
Standard Lufa soil^H^	-	-	2.2(0.1)^bc^	1.1(0.1)^b^	-	-

Concentrations in µg g^−1^ DW; standard deviation from five replicates in brackets. For soil characteristics (A-H) see Supplementary Table 2.

Significant differences between specific markers in soils are indicated by different letters. Forest organic - soil samples taken from organic layer of the soil; forest mineral – soil samples taken from uppermost mineral layer of the soil.

### Validation with soil, fungal, and synthetic melanin

**Fig. 5:**
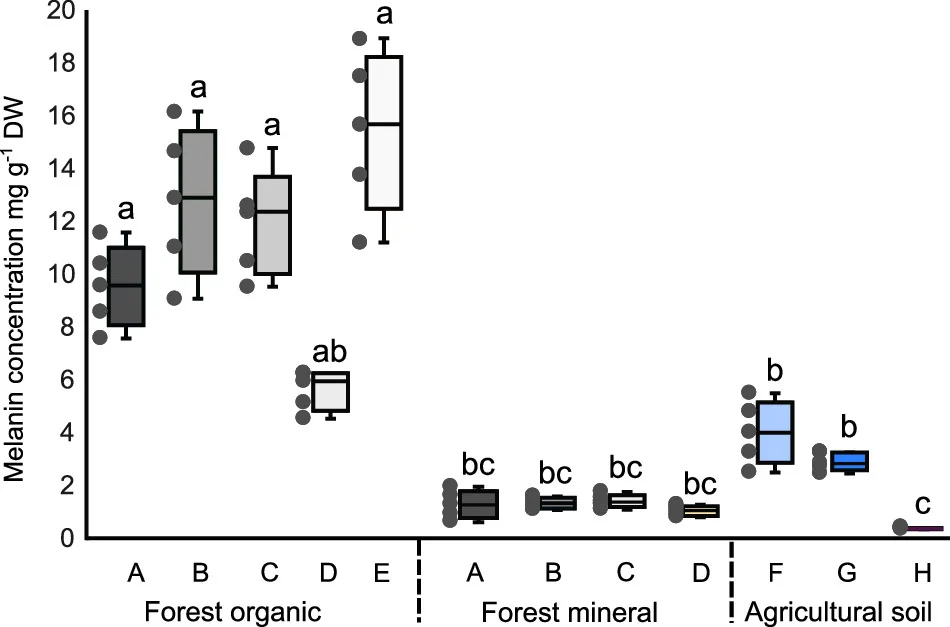
Concentrations of soil melanin measured with proposed protocol (sums of pheomelanin, eumelanin and allomelanin). Significant differences marked with different letters (Kruskal–Wallis test, *p* < 0.05). Box plots indicate median (middle line), 25th, 75th percentile (box) and 5th and 95th percentile (whiskers). *n* = 5. Soils from different sites (A-H) are marked with capital letters (OX axis), for soil characteristics see Suppl. Table 2. DW- dry weight.

Using the proposed method, we quantified melanin concentrations in fungi cultivated under laboratory conditions, in soils collected from forest sites, a forested peatland, and agricultural sites (Table 3)*. Mortierella* contained pheomelanin, *Cenococcum* contained allomelanin and *Tylospora* contained eumelanin. Synthetic eumelanin and *Sepia* melanin were rich in eumelanin markers, and allomelanin in respective allomelanin markers. Across the soil samples, allomelanin and eumelanin were the dominant forms of melanin in the soil samples, while pheomelanin was generally absent, except at very low concentrations in the forest soil at only two sites (Table 3). The highest concentration of total melanin (calculated as above for eumelanin and allomelanin) was detected in forested peatland soil and the organic layer of forest soils (see Fig. 5). The organic layer of forest soil with lower organic matter content had a lower melanin concentration, but the differences were not statistically significant. The lowest results were measured in agricultural soil.

### Correlations of melanin concentration with soil variables

**Fig. 6:**
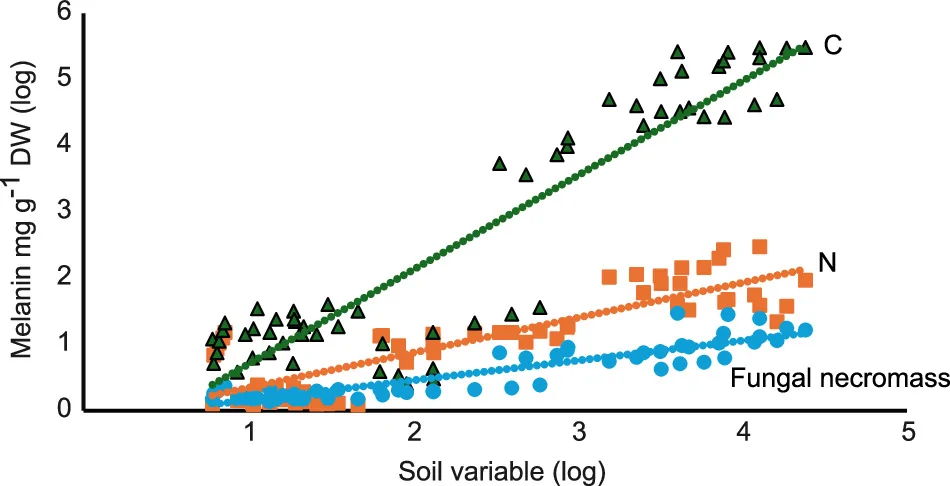
Correlations between soil melanin and soil properties. Correlation between soil melanin and soil variables, i.e., total C (green) total N (blue) and fungal necromass (orange). Correlations (Spearman test) plotted for the whole set of data.

We obtained significant positive correlations between the total soil melanin content measured with the proposed method and fungal necromass (*r* = 0.792, *p* < 0.001), total C (*r* = 0.777, *p* < 0.001) and total N content (*r* = 0.829, *p* < 0.001) across the analysed soil samples (see Suppl. Table 2 for soil characteristics). Compared with the fungal necromass, the melanin concentration was slightly more strongly correlated with C (*r* = 0.777 for melanin vs. *r* = 0.733 for necromass). Conversely, total N was more strongly correlated with necromass than with melanin (*r* = 0.847 vs. *r* = 0.829). The correlations between soil melanin and soil variables are plotted for the whole data set (Fig. 6) (see Suppl. Table 8 for the results of soil variables, i.e., fungal necromass, total C, and total N).

## Discussion

A key limitation in understanding fungal contributions to persistent SOC has been the inability to directly quantify fungal melanin in soil. Previous approaches relied primarily on genomic inference, preventing direct assessment of chemically preserved melanin pools within soil organic matter. By enabling direct quantification of fungal melanin in soil, our approach establishes fungal melanization as a measurable biogeochemical property rather than solely a microbial trait prediction.

In response to the proposed role of melanin–rich fungi in soil–microbial–plant interactions and soil C stabilization^7,14^, we developed an HPLC–based approach for precise qualitative and quantitative measurement of fungal melanin in soils. To quantify eumelanin and pheomelanin, we adapted and optimized an alkaline oxidation procedure^17^ which generates melanin–specific markers. For fungal allomelanin (1,8–DHN allomelanin), we established reliable oxidation products that serve as specific markers. Together, these developments enabled the quantification and identification of all three major types of fungal melanin within a single HPLC run in soil samples.

Our understanding of fungal melanin structure has evolved considerably over the past half–century. Early studies relied on alkali extraction of “melanin” from fungal biomass, followed by analyses that detected polysaccharides, proteins, lipids, nucleic acid derivatives and aromatic compounds in these extracts^22^, thus such defined melanins were very similar to soil humic acids^23^. Advances in analytical techniques have since enabled a more precise chemical definition of fungal melanins. We chemically define fungal melanins as allomelanin (derived from 1,8–DHN monomers), eumelanin (derived from DHI and DHICA), and pheomelanin (derived from 1, 4,-benzothiazine and 1, 4-benzothiazine carboxylic acid), none of which incorporate polysaccharides or proteins in their structures^21^. Proteins, carbohydrates, and lipids found in early melanin extracts are now understood to originate from the surrounding fungal cell wall rather than the melanin polymer itself^21,24^.

Plants can also produce allomelanin, an N–free melanin, but plant allomelanins are not 1,8–DHN derived^25^. Instead, plants typically use catechol, caffeic, chlorogenic, protocatechuic, or gallic acid^13^, and p–coumaric acid^26^ as precursors. Plant melanin synthesis is primarily associated with enzymatic browning following tissue damage, during which chloroplast–located polyphenol oxidase produces o–quinones that polymerize into allomelanin^13^. Some plant allomelanins also occur in seeds, but again they are not derived from the 1,8–DHN^13^. Given these differences, we evaluated the specificity of our method against plant melanins. We tested a wide range of plant materials potentially containing melanin–like compounds—pine and spruce seeds, cones, and roots, nut shells^13,27^, black oat grains^26^, and plant–derived polymers that do not contain melanin, such as spruce tannins and commercial lignin. The results clearly demonstrated that alkaline oxidation of these plant materials did not result in the production of fungal allomelanin markers (IPHA and PHA). The only detectable traces of allomelanin in plant roots corresponded to fungal presence, which was confirmed through analysis of ergosterol (a marker specific for fungal biomass) (Suppl. Table 5). Thus, our method is highly specific for fungal allomelanin.

We further compared our HPLC method with spectroscopic and spectrophotometric methods, which are faster and more cost–effective than HPLC; however, their reduced specificity often makes them less suitable for precise analyses in chemically complex soil matrices^28,29^. FTIR spectroscopy is widely used to characterize chemical compounds with unknown structures. It can distinguish the principal chemical classes in soil organic matter through the vibrational characteristics of their structural chemical bonds^30^. FTIR has been applied for the characterization of laboratory cultivated fungal melanin^20^ and to track chemical changes in fungal necromass following soil incubation in mesh bags^31^. We compared the FTIR spectra of synthetic melanin (eumelanin and allomelanin) with those of several plant–derived materials. The characteristic melanin bands overlapped substantially with the phenolic bands typical of lignin and spruce tannins, and similar features were also visible in the shells, cones, and seeds (Fig. 4), which did not show melanin markers in our HPLC method. More specifically, the FTIR bands characteristic of melanin, i.e., 3600–3000, 2858, 2927, 1650–1600 and 1300–1220 cm^−^^1^ (assigned as –OH and –NH stretching, aliphatic C–H stretching, aromatic C = C stretching, and C–N stretching, respectively^20,21^), overlap with these of fats, waxes, lipids (2920 cm^−^^1^), proteins (1650 cm^−^^1^), and phenolic compounds such as lignin and tannins (1650–1600, 1265 cm^−^^1^)^30^. This overlap is expected, as FTIR detects functional groups rather than polymer identity, and these groups are shared among lignin, tannins, and melanin. Thus, FTIR is not suitable for quantifying melanin in soil because of its lack of specificity. However, it may still serve as a useful, rapid screening tool for assessing the chemical features of pure fungal necromass obtained from laboratory cultures.

Alternatively, melanin concentration can also be measured with Azure A reagent^19^. Azure A is a cationic dye that binds to negatively charged cellular components (e.g., nucleic acids) making them visible under a microscope^32^. Owing to this broad affinity, the dye is not inherently specific to melanin. To overcome this issue, the Azure A protocol includes a pretreatment step in which all cellular components other than melanin are removed using concentrated acid (6 M HCl). We evaluated this method against a set of soil, fungal necromass, and synthetic melanin samples and a variety of plant–derived samples. The results clearly demonstrated a lack of specificity: Azure A produced equally high results for lignin, tannins, cones, and seeds (which do not contain fungal melanin) as it did for melanin–rich fungal necromass (see Fig. 3). Thus, we do not recommend the Azure A method for measuring melanin in soil samples.

We also examined alternative method for pheomelanin via reductive hydrolysis^33,34^, not tested before for soil samples. Instead of TTCA and TDCA, reductive hydrolysis with hydroiodic acid yields 4–amino–3–hydroxyphenylalanine (4–AHP) and 3–aminotyrosine (AT) as specific degradation products of pheomelanin^35^. However, reductive hydrolysis produces a higher background in soil extracts than alkaline oxidation, resulting in unreliable chromatograms. Considering this context and the need for separate sample preparation and HPLC runs for pheomelanin markers via reductive hydrolysis, we propose using our methodology, which relies on a single sample preparation (alkaline oxidation) and a single HPLC run, enabling reliable quantification of pheomelanin, eumelanin, and allomelanin.

Previous studies have suggested that fungal melanin may play a role in soil carbon (C) stabilization. Soil incubation experiments have shown that melanin slows the decomposition of fungal necromass^7,16^, and melanized fungal necromass has been proposed to contribute to more persistent soil organic C pools^36,37^. Similarly, DNA–based predictions of melanin in laboratory fungal incubations correlated well with soil C at sites where these species were abundant^11^. Using a proposed method to quantify soil melanin, we found strong positive correlations between soil melanin content and key soil variables associated with C stabilization, including fungal necromass, total C, and total N. These correlations were consistent across soils of diverse origins and characteristics, including highly organic forested peatlands, the organic layers of forest soils, and mineral soils from both forested and agricultural sites. Notably, the correlation between melanin and total C was even stronger than that observed for fungal necromass, suggesting that melanin content may provide complementary information regarding persistent fungal-derived C stabilization. This suggests that the chemical composition of fungal necromass, particularly melanization, may be more important for long–term soil C persistence than necromass quantity alone. Efforts to improve the predictive power of ecological models increasingly incorporate new variables, among which microbial necromass has emerged as an important factor in SOC stabilization^38–40^. Our findings indicate that including melanin measurements in such models could provide a more accurate indicator of C stabilization than fungal necromass alone, thereby enhancing predictions of soil C dynamics. More broadly, these results suggest that melanized fungal residues constitute a distinct and measurable pool of persistent microbial-derived soil C.

The soil concentration of eumelanin and pheomelanin could be overestimated due to input from animal residues, as mammals in their skin and hair and birds in their feather have melanin, also insects and soil animals may have melanin^41^. However, input of animal melanin is rather small compared to fungi due to biomass and differences in decomposition. The biomass of soil animals is far lower than soil fungal biomass^42,43^. Globally, biomass distribution is dominated by plants (450 Gt C) with a significant pool of fungal biomass (12 Gt C) and only 2 Gt C of animals (including humans)^44^. Thus, fungal residues provide an abundant and continuous source of melanin to the soil, whereas animal contributions are intermittent, variable, and significantly lower. Moreover, decomposition of animal residues (e.g., skin, hair and feathers) with eumelanin and/or pheomelanin could differ from decomposition of fungal necromass. Animal residues are in the litter layer, where they are exposed to UV which cause photodegradation and chemical modifications of melanin^45–47^ but most importantly, microbial decomposition is faster in the litter layer than in deeper layers of the soil^48^, where fungal necromass containing melanin is also deposited. In addition, fungal melanin is integrated into fungal cell walls, thus it is potentially more persistent in soil than animal eumelanin because of highly condensed aromatic structure and physical association with chitinous cell-wall matrices^16,49^. Thus, input of animal pheomelanin and eumelanin to the persistent soil melanin pool should be minimal compared to fungal melanin, moreover, main fungal melanin type, 1,8–DHN allomelanin, is not produced by animals^12^.

A potential limitation of our method could be the formulas used for converting alkaline oxidation markers into estimates of melanin concentration. To reflect naturally occurring melanin, we used *Sepia* melanin as a standard for eumelanin, and we propose allomelanin–rich *Cenococcum* fungi as a reference material for allomelanin. We advise always including such standards into analyses and calculations. Moreover, adsorption of melanin onto soil minerals may affect the efficiency of alkaline oxidation. Though we showed that % recovery is higher than 80% for mineral-bound melanin, we advise examining % recovery for each measured soil separately.

Further directions in soil fungal melanin research should also include investigation of how melanin–mineral interactions influence long-term carbon stabilization, fungal necromass persistence, and the ecological functions of melanized fungi in soils. Expanding studies on the diversity of fungal allomelanins, particularly among ectomycorrhizal and saprotrophic fungi, could substantially improve the understanding of C stabilization in the soil.

In conclusion, our work establishes fungal melanin as a directly measurable component of soil organic matter, enabling mechanistic investigation of melanized fungal necromass in soil C stabilization. This method provides opportunities to investigate how melanin influences soil carbon persistence and serves as a useful tool for studying fungal necromass stabilization and microbial stress responses. Moving fungal melanization from an inferred microbial property to a measurable biogeochemical variable enables the integration of fungal functional traits and necromass chemical quality into models of persistent soil C formation.

## Methods

### Standards

Standards of the pheomelanin and eumelanin oxidation products were bought from ChemPur company (ChemPur Feinchemikalien und Forschungsbedarf GmbH, Germany): PDCA (Cat no. AG008U96), PTCA (Cat no. AG006A0I), TDCA (Cat no. CRV824) and TTCA (Cat no. AG002V8B). The purity of the standards was confirmed with HPLC with UV detector and MS using the method described in this paper. The *m/z* values of PDCA, PTCA, TTCA and TDCA reflected those described earlier^50^ for laboratory–prepared standards. Phthalic acid and isophthalic acid were purchased from Sigma–Aldrich (St Luis, USA) (Cat no. 8.2298.0100 and A14445.30, respectively). Synthetic eumelanin (Cat no. M8631), *Sepia officinalis* melanin (Cat no. M2649) and the monomer (1,8–dihydroxynaphthalene, 1,8–DHN; Cat no. 740683) used to produce synthetic allomelanin were obtained from Sigma–Aldrich.

### Synthetic allomelanin

Synthetic allomelanin (1,8–DHN allomelanin) was prepared by the autooxidation of 1,8–DHN using a previously published protocol, similar to the protocol used to produce DOPA–melanin^51,52^. For this purpose, 100 mg of 1,8–DHN was added to 20 ml of 0.01 M NaOH, mixed for 48 h and bubbled with air. Afterwards, 1 ml of 0.5 M HCl was added, and centrifugation (5000 g, 15 min) was performed to pellet the insoluble melanin. The supernatant was removed, and the precipitate was washed ten times with 0.01 M HCl and then four times with water, followed by cleaning with VWR centrifugal filters with a 10 K MWCO and washing another three times with water. Finally, the synthetic allomelanin was freeze–dried.

### Detailed protocol

Samples for measurements of the quality and quantity of melanin need to be freeze–dried. For melanin measurements from fungal necromass, we recommend 2 to 5 mg; for the organic layer of the soil and peat soils, we recommend 50 mg of dried material; and for the mineral layer of the soil, we recommend 250 mg. These amounts of material were weighed into glass test tubes (volume of 12 mL) with Teflon caps. Then, 2 mL of 6 M HCl was added, and the samples were incubated at 80 °C in a heat block (Dri–Block, TECHNE, UK) for 1 h (most optimal conditions of temperature and HCl incubation time, see Suppl. Table 3 and 4).

After cooling, the samples were centrifuged (2500 × g, 5 min, Eppendorf centrifuge, model 5804, Germany), and the supernatant was removed. The precipitate was washed twice with ultrapure water (2 mL), and the supernatant was removed after centrifugation (2500 × g, 5 min). The precipitate was then subjected to alkaline oxidation. The alkaline oxidation procedure was as follows: 200 µL of ultrapure water was added to the precipitate, followed by 750 µL of K_2_CO_3_ (1 M) and 50 µL of H_2_O_2_ (30%). The samples were incubated for 4 hours in the dark with vigorous shaking (250 rpm on an orbital shaker; Braun Biotech Certomat R, Switzerland). Immediately after incubation, 100 µL of Na_2_CO_3_ (10%) was added to remove excess H_2_O_2_, and 280 µL of 6 M HCl was added. After centrifugation (2500 × g, 5 min), 400 µL of the supernatant was transferred to another glass test tube and evaporated to dryness under a stream of pure N_2_ at 40 °C (in a heat block). The dry material was dissolved in 10% methanol with 0.1% trifluoroacetic acid (TFA). The samples were then filtered through a 0.2 µm PTFE filter into HPLC vials with inserts (VWR, Cat no. 548–0425). For standards, a mixture of PTCA, PDCA, TTCA, TDCA, PHA and IPHA was mixed in 10% methanol with 0.1% TFA. The samples were run on a HPLC (Arc HPLC Waters with a UV detector and qDa Waters MS, Waters, Milford, MA, USA) with a C18 reverse–phase column (Kinetex 5 µm C18 100 Å, 4.6 × 250 mm; Phenomenex, Torrance, CA, USA) at 25 °C in a column oven with a flow rate of 0.4 mL min^−^^1^. Gradient elution consisted of pure methanol with 0.1% TFA and water with 0.1% TFA (as shown in Table 4). To avoid retention loss in the column due to the high water content (90%) observed for C18 columns^53^, we suggest always conditioning the column with 10 min 0.4 mL min^−^^1^ 100% eluent A (100% methanol with 0.1% TFA) as first run in the batch, followed by “standard zero” (10% methanol with 0.1% TFA), followed by a set of standards and samples, and finally, column conditioning with 10 min run with eluent A. This method provides the most precise results, with no retention loss owing to a low concentration of organic solvent^53,54^. Detection was performed with a UV detector at 270 nm (Waters). The column effluent was directed not only to the UV detector but also to the mass detector with an isocratic solvent pump manager (ISM, Waters). A post–column solvent consisting of 50% acetonitrile with 0.5% formic acid (0.1 mL min^−^^1^) was used to improve the ionization of the samples with no disturbance to the separation. The peak purity was confirmed with an MS (Acquity qDa, Waters) with electrospray ionization (ESI) in positive and negative mode with a cone voltage of 15 V, a probe temperature of 600 °C and a capillary voltage of 0.8 kV. The mass scans were collected in the *m/z* range of 120–220 in both negative and positive mode. An HPLC with a MS mass detector was run on EMPOWER 3.6 software (Waters, USA). The peak areas and mass–to–charge ratios were calculated with ACD Labs software.

**Table 4:** Conditions of gradient elution

Time, min	% of eluent A	% of eluent B
0	10	90
20	10	90
30	95	5
35	95	5
40	10	90
42	10	90

### Soil sampling

We gathered soil samples from a set of study sites with various characteristics ranging from very high clay content agricultural sites to low clay forest sites and forested peatlands (for the soil characteristics, see Suppl. Table 2). Soil was sampled from control plots in field studies (five plots), visible litter was removed, and samples were sieved at the site via a 2 mm sieve (except forested peatland) and transported in cold boxes to the laboratory, where they were immediately freeze–dried and finely milled. From the forested peatland, samples were taken from 0 to 10 cm layer; for the other sites, samples were taken from the organic layer and uppermost mineral layer of the soil (except agricultural soil). Standard Lufa soil (v2.2) was ordered from Lufa Speyer, Germany.

### Soil minerals

The soil minerals were purchased from Nanochemazone (Alberta, Canada) and these include illite (Cat no. NCZ–NP–844/25), kaolinite (NCZ–NP–624/24), goethite (NCZ–NP–404/23), ferrihydrite (NCZ–NP–405/23), representing clay and iron (oxyhydr)oxide minerals. These minerals were washed 4 times with ultrapure water until the pH was around neutral and dried. The interaction with melanin was studied as follows: 100 mg of minerals were mixed with 0.5 mL of 10 mM CaCl_2_ to which alkaline oxidation markers or melanin (synthetic eumelanin, allomelanin, *Sepia* melanin, *Cenococcum* necromass) were added. After overnight mixing on orbital shaker (200 rpm) samples were exposed to alkaline oxidation followed by estimation of % recovery (Suppl. Table 7).

### Fungal necromass

Fungi were obtained from the Natural Resources Institute Finland (Luke) culture collection (*Tylospora asterophora* R–SP01 and *Cenococcum geophilum* R–FC03), and *Mortierella* sp. D–081 305 was obtained from VTT culture collection. Fungal mycelia were collected from cellophane membranes placed on top of modified Melin–Norkrans nutrient medium and crushed into liquid growth media with an OMNI mixer. The prepared fungal strains were subsequently grown in 500 mL Erlenmeyer flasks in liquid–modified Melin–Norkrans nutrient medium (22 °C, 100 rpm) for 3–6 months. The mycelium was rinsed with ultrapure water, collected on a 2 mm sieve, lyophilized, and stored at 4 °C.

### Azure A method

Melanin content was measured with Azure A for determining fungal necromass^16,19^. Briefly, samples were treated with 6 M HCl at 80 °C for four days and later washed four times with 10 ml of water to remove HCl. The samples were mixed with Azure A dye (dissolved in 0.1 M HCl) and incubated for 90 minutes. The absorbance was measured at 610 nm. Melanin concentrations were calculated on the basis of the difference in absorbance of the Azure A dye solution before and after exposure to melanin from the samples. *Sepia* melanin was used as a standard.

### Fourier–transform infrared spectroscopy

We used Fourier–transform infrared spectroscopy with attenuated total reflectance (FTIR–ATR; Shimadzu IRPrestige–21 FTIR 260 spectrophotometer, Kioto, Japan; Specac Quest diamond ATR accessory, Orpington, UK). We acquired the spectra at a resolution of 4 cm^−^^1^ by averaging 50 scans per sample over the range of 4000–400 cm^−1^ with atmospheric corrections (CO_2_, H_2_O, and smoothing at level 7)^55^.

### Ergosterol and microbial necromass

Fungal biomass was measured through its biomarker, ergosterol, with LC–MS equipped with reversed–phase column (Phenomenex C18 150 × 4.6 mm, 100 Å)^56^. Ergosterol was extracted from 0.25 g samples (freeze–dried and milled) by adding 1 mL cyclohexane and 4 mL 10% KOH in methanol. After 15 min ultrasonication, the samples were incubated at 70 °C for 1 h. Further, 1 mL water and 2 mL cyclohexane were added, and the samples were shaken, and the cyclohexane phase was moved to the next test tube. The procedure with water and cyclohexane was repeated and the combined extracts were evaporated under nitrogen gas at 40 °C in a heat block. The samples were re–dissolved in 100% methanol and filtered (0.2 μm PTFE filter, Phenomenex). Ergosterol was analysed using a 10 μL sample injection and isocratic separation with 100% methanol (1 mL min^−1^) and detected at 282 nm and with mass detector using electrospray ionization in positive ion mode and selected ion recording to detect and quantify ergosterol. Ergosterol results were converted into fungal biomass using Eq. 4 as proposed in ref. ^57^:

4 $${{{\rm{FB}}}}\left({{{\rm{\mu g\; g}}}}-1{{{\rm{DW}}}}\right)={{{\rm{ergosterol}}}}\left({{{\rm{\mu g\; g}}}}-1{{{\rm{DW}}}}\right){xfxRf}$$

where f = 250 (1/4 ×1000, mg biomass µg^−1^ ergosterol) and Rf = 1.61 (correction factor for average recovery percent, 1/0.62).

Microbial necromass was studied via its marker, the amino sugar glucosamine for fungal necromass and muramic acid for bacterial necromass^58^. Briefly, freeze–dried samples were washed with 0.2 M NaOH to remove unbound amino acids and amino sugars. The samples were then washed with water to remove the NaOH and hydrolyzed with 6 M HCl (in a heat block for 7 h at 100 °C). After centrifugation, 100 µL of supernatant was evaporated dryness under a stream of nitrogen at 40 °C and 100 µL of ultrapure water was added and 100 µL internal standard (homocysteic acid, 10 µM) and then 50 µL of borate buffer (0.5 M, pH 9.3) and 250 µL of fluorenylmethyloxycarbonyl chloride (FMOC, 7.5 mM dissolved in acetone). After 10 minutes of incubation at room temperature, 1 mL of heptane was added, and samples were centrifuged (2500 g × 5 min). Lower phase was filtered (0.2 µm PTFE, Phenomenex, USA) into HPLC vials. Amino sugars derivatized with FMOC were separated using HPLC (Arc Waters, USA) equipped with ODS Hypersil column (5 µm, 250 × 4.6 mm, Hewlett–Packard, USA) at temperature of 25 °C and flow rate of 0.7 mL min^−1^. We used gradient elution with buffer (40 mM ammonium acetate buffer, with 0.1% triethylamine, pH 5.7) and 100% methanol. FMOC–amino sugars were measured with a fluorescence detector (Waters, USA) with excitation 260 nm and emission 330 nm. As an external standard, commercial amino sugars in various concentrations were used.

### Melanin–like compounds and materials

The black oat (*Avena sativa L*.) grains were obtained from commercial sources (Agribioshop, Italy). Roots, seeds and cones of pine and spruce were obtained from the experimental site located next to the SMEAR II station near the Hyytiälä Forest Station of the University of Helsinki, in Juupajoki, Finland (61°51’N, 24°17’E). Condensed tannins were extracted from spruce roots (obtained from above mentioned site) and purified as described previously in ref. ^59^.

Lignin (commercial) was obtained from Sigma–Aldrich (Cat no. 471003). Nut shells were obtained from local commercial shop.

### Total C and N of soil samples

The total C and total N from the soil samples were measured with a CN elemental analyser (LECO TruMac CN, LECO Corporation, USA).

### Statistics

Differences in melanin markers among soils (Table 3) and melanin concentrations measured using Azure A (Fig. 3) were assessed using one–way analysis of variance (ANOVA) followed by Tukey’s post hoc test. Normality and homogeneity of variances were evaluated using the Shapiro–Wilk and Levene’s tests, respectively, and the data were log₁₀–transformed when necessary to meet ANOVA assumptions. Differences in summed melanin content and its relationships with soil variables (Fig. 5, Suppl. Table 8) were analysed using the Kruskal–Wallis test due to violations of ANOVA assumptions. Correlations were assessed using Spearman’s rank correlation. All the statistical analyses were performed using SPSS (v.30). A significance threshold of *P*  <  0.05 was applied, and significant differences are indicated by different letters.

### Reporting summary

Further information on research design is available in the Nature Portfolio Reporting Summary linked to this article.

## Supplementary information

supplementary information

Reporting Summary

Transparent Peer Review file

## Source data

Source Data

## Data Availability

The data generated in this study are provided in the Supplementary Information and in Source Data file. Source data are provided with this paper.
