# Supplementary material for: A method quantifying soil melanin to understand fungal impacts on soil carbon stability
Source: Nat Commun. 2026 Oct 11;17:11524. doi: 10.1038/s41467-026-78616-4 (PMC13745510; doi:10.1038/s41467-026-78616-4)
Supplement: Supplementary file 1 [file 41467_2026_78616_MOESM1_ESM.pdf]

Supplementary Information for

A method quantifying soil melanin to understand fungal impacts on soil carbon stability

Authors: Sylwia Adamczyk[1], Bartosz Adamczyk[1]*

[1]Affiliation: Natural Resources Institute Finland (Luke), Latokartanonkaari 9, FI-00790 Helsinki, Finland

*Corresponding author email: bartosz.adamczyk@luke.fi

Orcid:

Sylwia Adamczyk: 0000-0002-9051-9302

Bartosz Adamczyk: 0000-0003-4286-9508

Phthalic acid (PHA) and isophthalic acid (IPHA) as alkaline oxidation products of fungal allomelanin

Fungal allomelanin is a polymer of 1,8-DHN. To prove that phthalic acid and isophthalic acids may be produced from fungal allomelanin under alkaline oxidation conditions, in line with the proposed protocol, we compared the retention times of the oxidation products of synthetic 1,8DHN-allomelanin with the retention times of phthalic acid and isophthalic acid, which were the same. We also compared the *m/z* values of allomelanin alkaline oxidation products in both the positive and negative ionization modes with those of PHA and IPHA. These were identical. After alkaline oxidation, the monomer of fungal allomelanin, i.e., 1,8-DHN, produced peaks with the same retention times and *m/z* values as IPHA and PHA (Supplementary Fig. 1). Similarly, also *Cenococcum* fungal samples (producing 1,8DHN-allomelanin) after alkaline oxidation produced IPHA and PHA markers (the same retention time and *m/z* values). Moreover, IPHA and PHA were also found in soil samples (see Fig. 1).

We also studied other potential markers of allomelanin with chemical structures similar to those of allomelanin monomer. Below are examples of compounds, other than PHA and IPHA, that could potentially serve as markers of 1,8-DHN allomelanin; however, their *m/z* values and retention times precluded their use for this purpose (Supplementary Table 1).

Supplementary Table 1. Examples of other compounds studied as potential markers of allomelanin alkaline oxidation products.

Compound	Retention time, min	*m/z*, positive ion mode	*m/z*, negative ion mode
5-Hydroxy-p-naphthoquinone	37.5	158, 135, 143, 175	155, 205, 135, 167
2-Hydroxy-1,4-naphthoquinone	36.3	175, 197, 158	155, 152, 154, 170, 205
5,8-Dihydroxy-1,4-naphthoquinone	38.3	159, 171, 131, 125, 163, 167	154, 166, 135, 139, 152
2-methyl-1,4-naphthoquinone	38.1	158, 136, 135, 214, 149	154, 138, 204, 205, 138

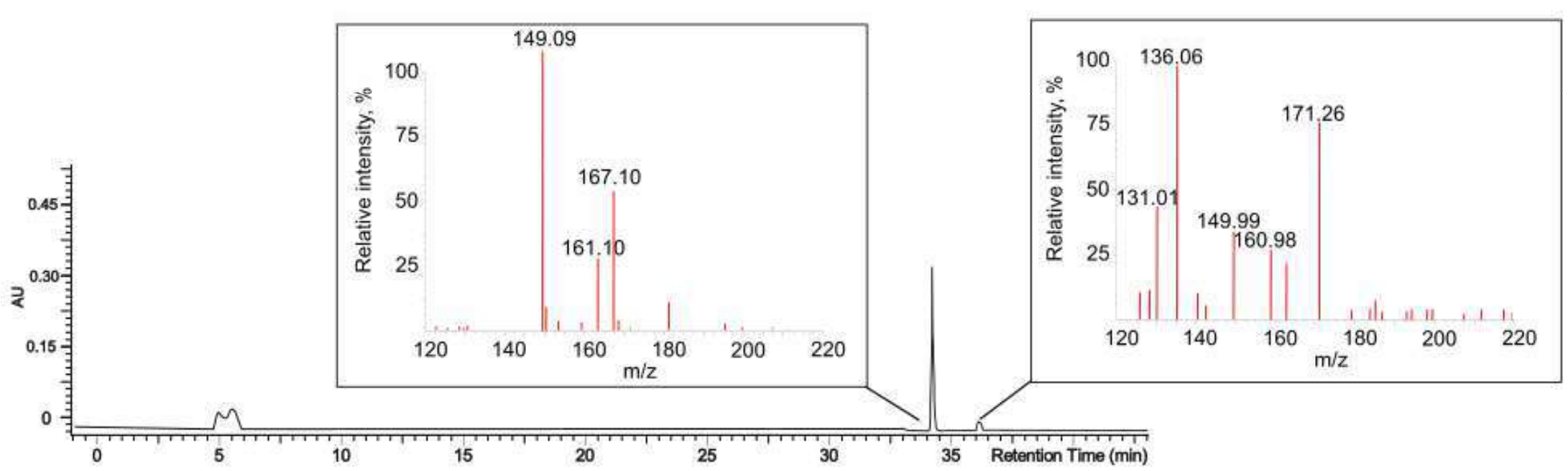

Supplementary Fig. 1. Chromatogram of the alkaline oxidation of 1,8-DHN with mass scans in positive ion mode.

Supplementary Table 2. Characteristics of soil samples. For more description of the sites see cited literature.

	Forest site[A] [2]	Forest site[B] [3]	Forest site[C] [4]	Forest site[D] [4]	Forested peatland[E] [5]	Agricultural site[F6]	Agricultural site[G] [7]	Lufa standard soil[H] [8]
Latitude								
	62.54°N	61.84°N	66.20°N	61.51°N	61.10°N	60.80°N	40.53°N	49.31°N
Longitude								
	24.34°E	24.26°E	26.40°E	29.22°E	25.15°E	23.48°E	3.30°W	8.32°E
annual mean temperature, °C								
	4.3	3.5	0.7	4.4	4.8	7.3	15	10
annual mean precipitation, mm								
	498	711	614	723	644	713	450	700
dominant plant								
	Pinus sylvestris	*P. sylvestris*	*Picea abies*	*Betula pendula*	*P. abies*	*Hordeum vulgare*	*H. vulgare*	meadow species
ground vegetation								
	Vaccinium vitis-idaea, Calluna vulgaris	*V. vitis-idaea C. vulgaris*	*V. vitis-idaea V. myrtillus*	*V. vitis-idaea V. myrtillus*	*V. myrtillus*	-	-	*Lolium perenne, Phleum pratense, Lolium perenne*
parent material								
	glacial till	glacial till	glacial till	glacial till	glacial till and peat deposits	fine-textured sediments	alluvial sediments	alluvial sediments
soil type								
	fine sand	haplic podzol	podzol	podzol	peat deposits	Cambisol	Luvisols	Fluvisol
soil texture								
	loamy sand	silty loam	loamy sand	fine sand till	Fibric/hemic peat	clay rich	balanced sand, silt, and clay	sandy loam
clay content, %								
	15	4	5	17	<3	32	39	12
organic matter, %, organic layer								
	52	65	85	31.5	>90% (no mineral layer)	5 (no organic layer)	2 (no organic layer)	4

organic matter, % mineral layer								
	5	3	2-5	2-5	-	-	-	-
humus type								
	mor	mor	mor	mor	-	-	-	mull
organic layer thickness								
	4.3	3	2-5	4.2	-	-	-	30
CEC, cmolc kg^{-1}								
	20-30	20-30	20-30	5-10	30–70	15	15	9.8
pH, organic layer								
	4.5	4	3.8	4.0	4.5	5.2	7.8	5.65
pH, mineral layer								
	3.7	3.7	3.5	3.5	-	5.2	7.8	5.65
C/N ratio organic layer								
	43	28	30	19.5	35	11.5	8.75	14
C/N ratio mineral layer								
	30	12	20	10	-	11.5	8.5	14

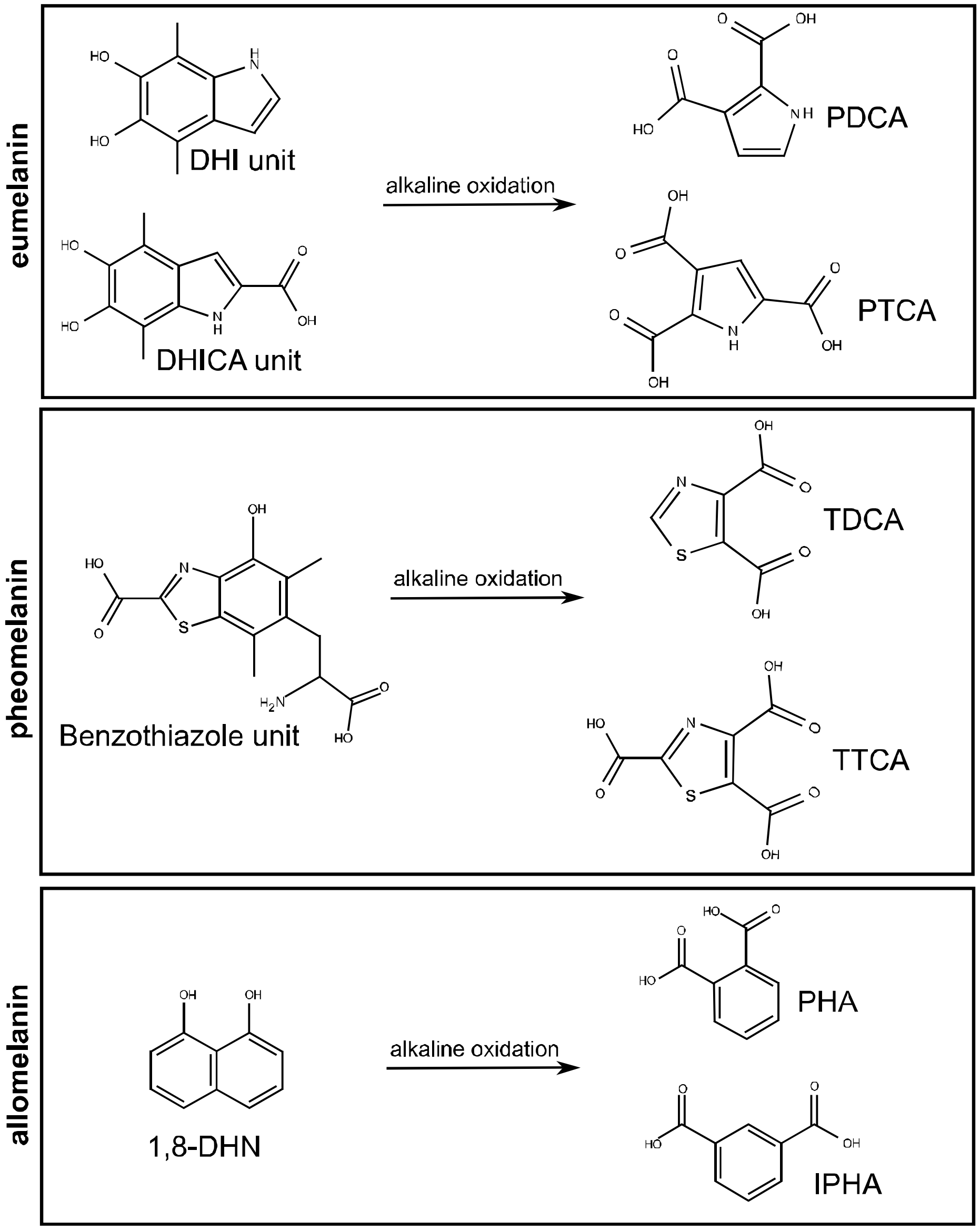

Supplementary Fig. 2. Chemical structures of monomers of eumelanin, pheomelanin and 1,8DHN-allomelanin and their respective products of alkaline oxidation: pyrrole-2,3-dicarboxylic acid (PDCA), pyrrole-2,3,5-tricarboxylic acid (PTCA), thiazole-4,5-dicarboxylic acid (TDCA) and thiazole-2,4,5-tricarboxylic acid (TTCA). Eumelanin consists of DHI and DHICA units, DHI - 5,6-dihydroxyindole, DHICA - 5,6-dihydroxyindole-2-carboxylic acid. Allomelanin: 1,8-dihydroxynaphthalene, (1,8-DHN), PHA – phthalic acid, IPHA – isophthalic acid.

The *m/z* values and key fragment ions of all the standards (obtained using ACD/MS Fragmenter (ACD/Labs, Toronto, Canada) can be found below (Supplementary Figs. S3–S8).

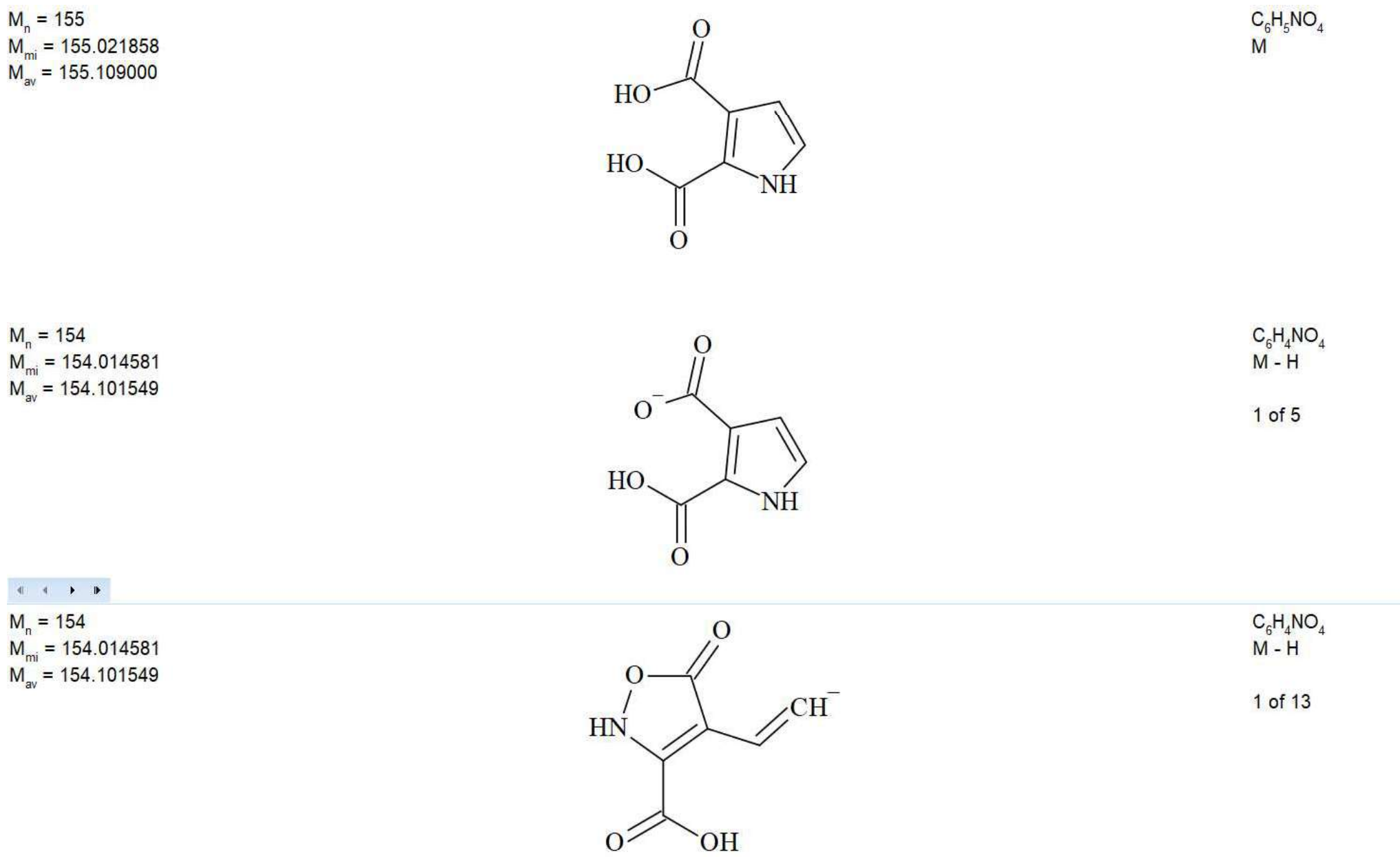

Supplementary Fig. 3. Key fragment ions of PDCA observed in the mass spectrum (ESI-).

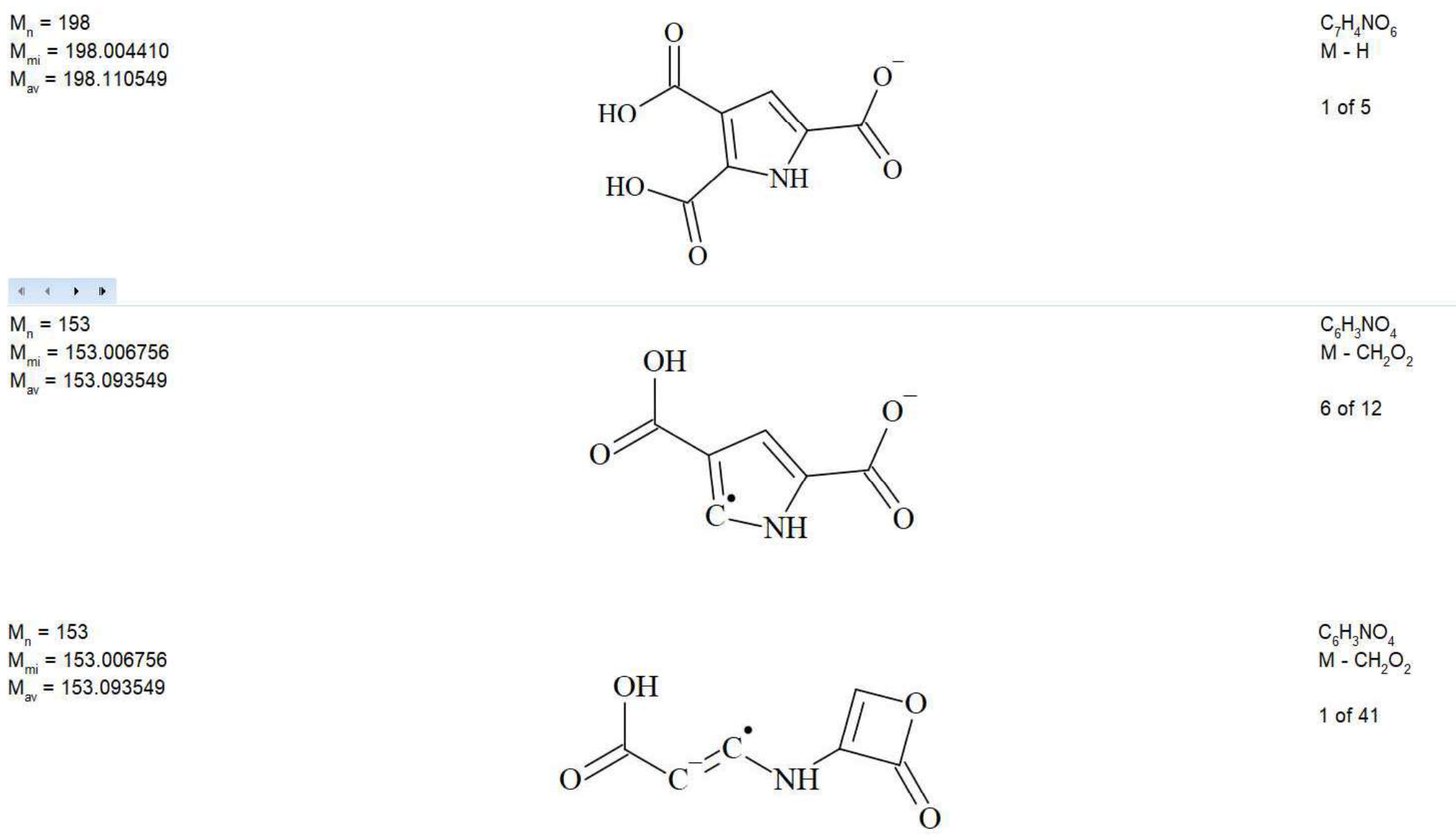

Supplementary Fig. 4. Key fragment ions of PTCA observed in the mass spectrum (ESI$^-$).

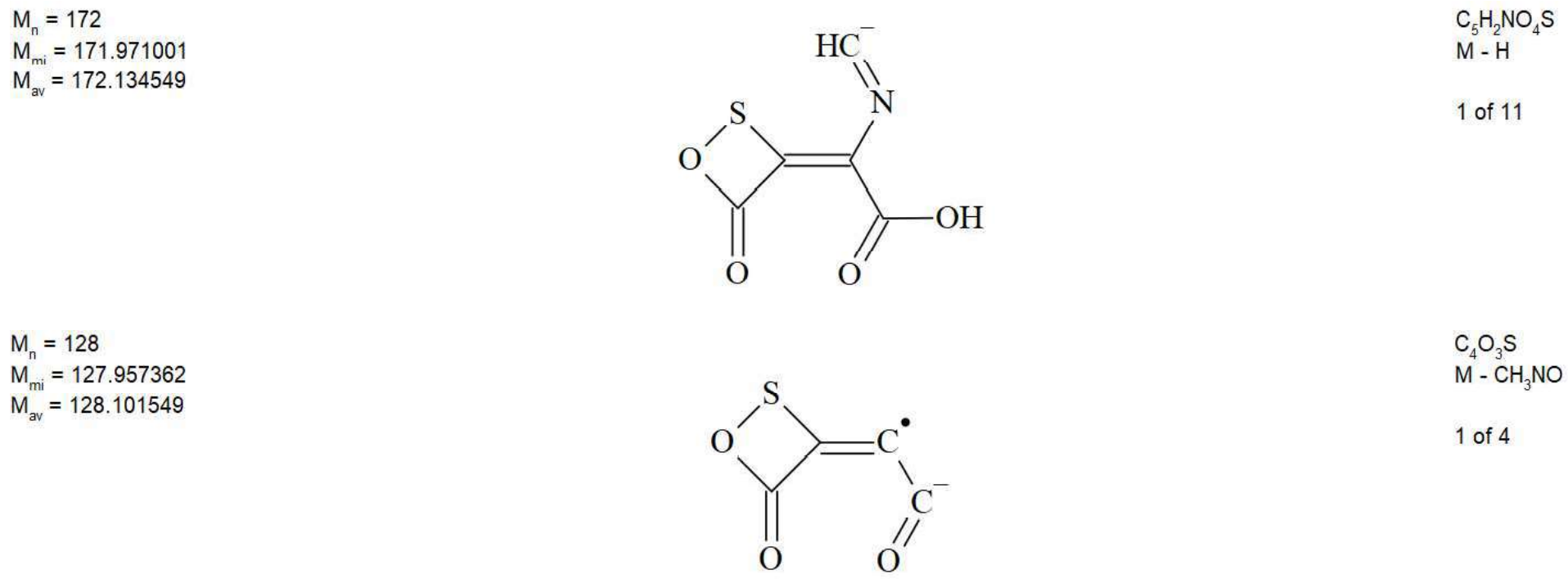

Supplementary Fig. 5. Key fragment ions of TDCA observed in the mass spectrum (ESI^{-}); the ion at *m/z* 205 likely corresponds to [M+Cl]$^{-}$.

M_n = 128
M_{mi} = 127.981172
M_{av} = 128.125549

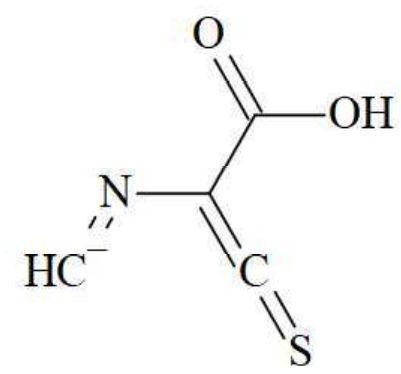

$C_4H_2NO_2S$
M - C_2HO_4

1 of 2

M_n = 172
M_{mi} = 171.971001
M_{av} = 172.134549

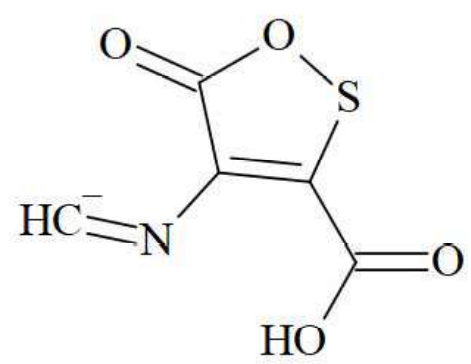

$C_5H_2NO_4S$
M - CHO_2

1 of 6

Supplementary Fig. 6. Key fragment ions of TTCA observed in the mass spectrum (ESI$^-$).

M_n = 167
M_{mi} = 167.033885
M_{av} = 167.139451

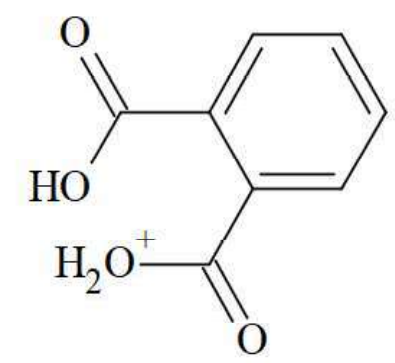

$C_8H_7O_4$
M + H

1 of 2

M_n = 167
M_{mi} = 167.033885
M_{av} = 167.139451

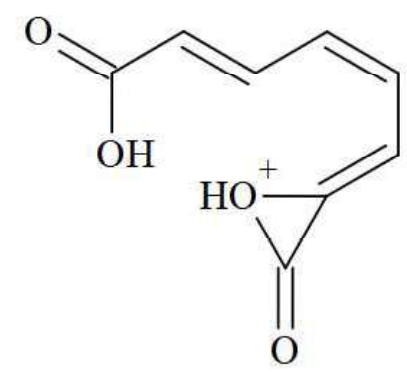

$C_8H_7O_4$
M + H

1 of 8

M_n = 149
M_{mi} = 149.023320
M_{av} = 149.124451

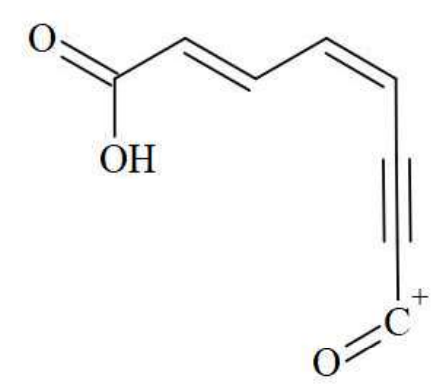

$C_8H_5O_3$
M - HO

1 of 43

M_n = 171
M_{mi} = 171.005265
M_{av} = 171.106221

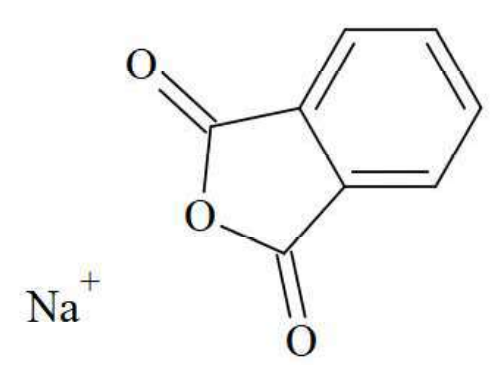

$C_8H_4NaO_3$
M + Na - H_2O

1 of 2

M_n = 161
M_{mi} = 161.020915
M_{av} = 161.111221

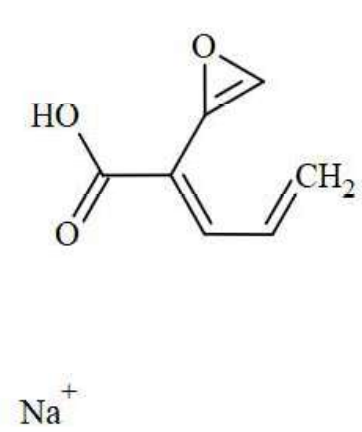

$C_7H_6NaO_3$
M + Na - CO

2 of 3

Supplementary Fig. 7. Key fragment ions of PHA observed in the mass spectrum (ESI^+).

M_n = 171
M_{mi} = 171.005265
M_{av} = 171.106221

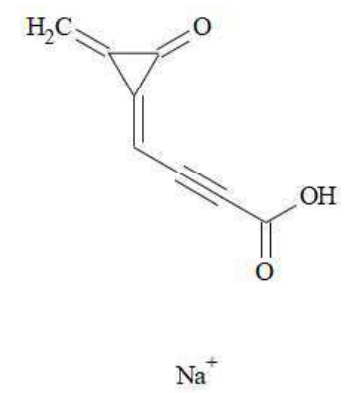

$C_8H_4NaO_3$
M + Na - H_2O

1 of 12

M_n = 150
M_{mi} = 150.031145
M_{av} = 150.132451

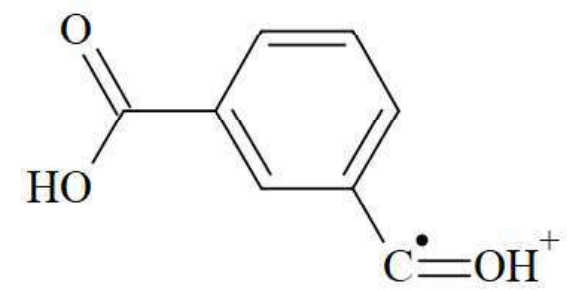

$C_8H_6O_3$
M - O

M_n = 167
M_{mi} = 167.033885
M_{av} = 167.139451

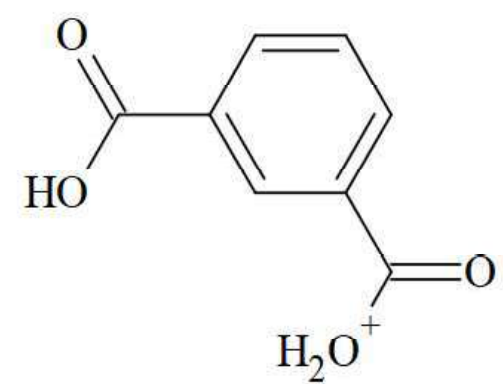

$C_8H_7O_4$
M + H

1 of 2

M_n = 161
M_{mi} = 160.984530
M_{av} = 161.067221

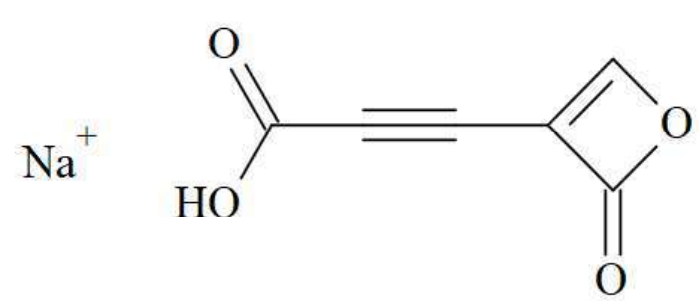

$C_6H_2NaO_4$
M + Na - C_2H_4

1 of 3

M_n = 136
M_{mi} = 136.015495
M_{av} = 136.105451

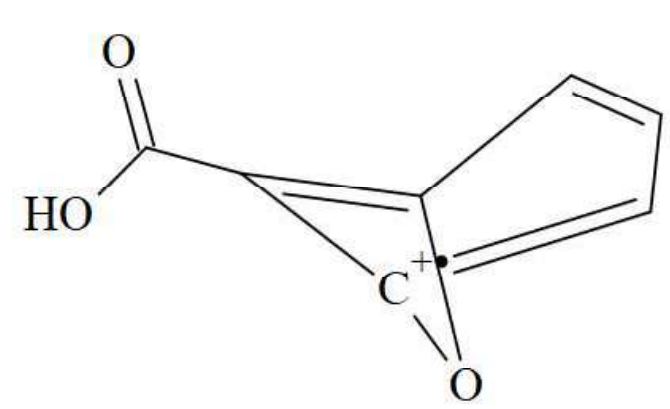

$C_7H_4O_3$
M - CH_2O

1 of 3

M_n = 131
M_{mi} = 131.012756
M_{av} = 131.109451

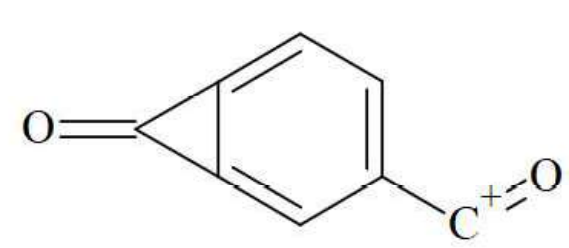

$C_8H_3O_2$
M - H_3O_2

1 of 4

Supplementary Fig. 8. Key fragment ions of IPHA observed in the mass spectrum (ESI$^+$).

Reductive hydrolysis to obtain pheomelanin markers

Reductive hydrolysis was conducted as described in[10]. Briefly, the samples (fungi and soil) were exposed to 30% phosphinic acid (H_3PO_2) and 57% hydriodic acid at 130 °C for 20 h. Hydrolysate was evaporated to dryness and the residue was redissolved in 0.1M HCl. For the separation and quantitation we followed LC-MS protocol[11], on the same equipment which we used for other melanin markers to be able to estimate peak purity *via* mass spectrometry. We used ZIC-HILIC column (250 mm, I.D. 2.1 mm, particle size 3.5 µm), mobile phase consisted of acetonitrile and 0.1 M ammonium acetate buffer, pH 4.5 (82:18, v/v), flow rate 0.2 mL min^{-1}, column temperature 25 ºC. The column effluent was directed not only to the UV detector but also to the mass detector with an isocratic solvent pump manager (ISM, Waters). A post–column solvent consisting of 50% acetonitrile with 0.5% formic acid (0.1 mL min^{-1}) was used to improve the ionization of the samples with no disturbance to the separation.

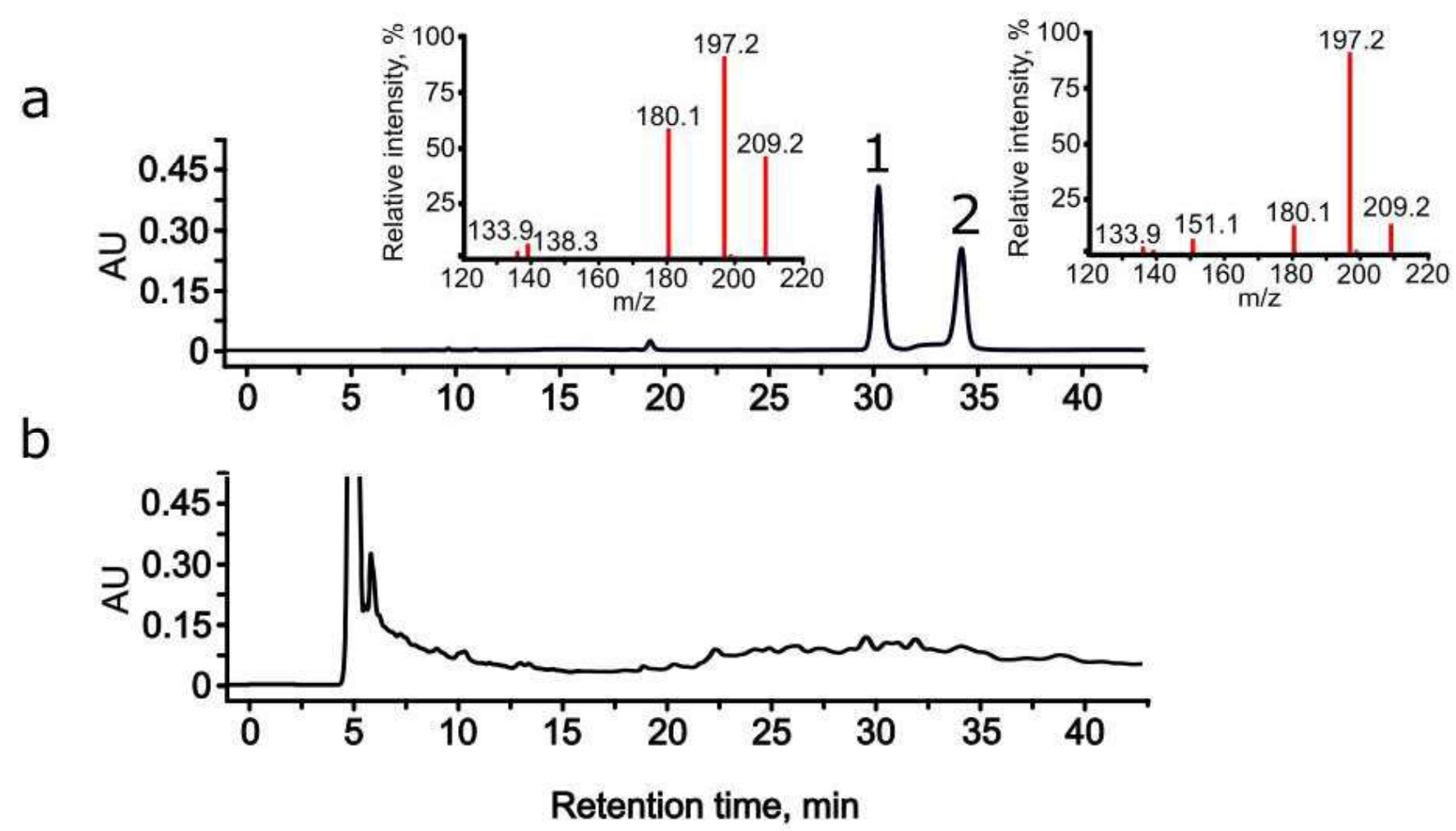

Supplementary Fig. 9. **Chromatograms of products of reductive hydrolysis**. **a** standards: 1 – 4-amino-3-hydroxyphenylalanine (AHP), 2 – 3-aminotyrosine (AT), with their mass scans **b** – example of soil sample (Forest organic soil[A]). The ion at *m/z* 197 corresponds to molecular ion of 4-AHP and AT $[M+H]^+$. The ions *m/z* at 180 and 151 correspond to $[M+H\text{-}NH_3]^+$ and $[M+H\text{-}HCOOH]^+$, *m/z* at 133.9 corresponds to $[M+H\text{-}CH_4NO_2]^+$ and *m/z* at 138 $[M+H\text{-}C_2H_5NO]^+$ and *m/z* at 209 corresponds to $[M+H+C_2H_4\text{–}O]^+$.

Supplementary Table 3. Percentage of melanin marker concentrations as affected by length of incubation with 6M HCl. 100% values presented in Table 3. Average of 4 replicates with standard deviation. Soil characteristics in Supplementary Table 2.

sample	no HCl	1h HCl	2h HCl	4h HCl	8h HCl	12h HCl	24h HCl
melanin standards							
Sepia melanin	75(2.9)	100(2.1)	101(1.5)	96(0.8)	90(0.9)	80(4.1)	69(5.7)
synthetic eumelanin	80(3.0)	101(0.9)	100(1.4)	95(1.8)	92(1.8)	82(5.2)	68(6.4)
synthetic allomelanin	81(2.4)	99.5(1.4)	100(0.8)	92(2.4)	91(2.5)	86(3.1)	70(4.7)
fungi							
Cenococcum	66(3.1)	101(1.7)	99(1.4)	93(2.1)	88(2.9)	82(2.4)	72(3.7)
Tylospora	68(2.5)	102(2.5)	98(1.7)	92(2.4)	87(2.8)	83(3.0)	70(2.4)
soils							
Forest organic[A]	70(2.8)	100(0.9)	100(1.5)	95(1.7)	90(2.3)	86(2.7)	74(3.8)
Forest mineral[B]	75(1.8)	99.4(1.2)	100(0.7)	94(1.2)	87(2.7)	84(3.7)	70(2.6)
Forested peatland[E]	77(0.8)	101(1.5)	98(1.7)	93(1.6)	86(3.1)	80(2.7)	68(3.4)
Lufa soil[H]	79(1.1)	102(1.9)	99(2.1)	95(1.7)	90(3.4)	86(1.4)	67(2.0)

Supplementary Table 4. Percentage of melanin marker concentrations as affected by temperature of incubation with 6M HCl (for 1h). 100% values presented in Table 3. Average of 4 replicates with standard deviation. Soil characteristics in Supplementary Table 3.

sample	25ºC	40ºC	60ºC	70ºC	80ºC	90ºC	105ºC
melanin standards							
Sepia melanin	75(1.2)	76(2.1)	79(2.0)	90(1.1)	100(1.1)	98(1.4)	94(1.8)
synthetic eumelanin	77(0.7)	79(0.8)	85(0.9)	92(1.5)	101(1.0)	97(1.5)	94(4.2)
synthetic allomelanin	78(0.9)	79(0.7)	88(1.6)	91(1.0)	99(0.8)	97(0.8)	92(1.8)
fungi							
Cenococcum	60(2.5)	66(4.1)	75(1.5)	85(1.4)	101(1.4)	96(1.8)	92(4.2)
Tylospora	68(3.1)	69(2.5)	78(2.8)	88(0.5)	99(0.7)	95(0.8)	90(1.7)
soils							
Forest organic[A]	59(2.1)	62(0.9)	78(1.8)	88(1.7)	99(1.1)	98(1.5)	92(3.3)
Forest mineral[B]	67(2.9)	70(2.7)	79(2.1)	88(0.8)	98(2.1)	97(2.0)	91(3.2)
Forested peatland[E]	66(4.1)	68(1.9)	81(1.5)	89(0.9)	100(1.4)	97(3.0)	92(3.8)
Lufa soil[H]	68(2.7)	71(1.4)	84(1.6)	91(0.4)	101(1.3)	98(1.9)	91(2.4)

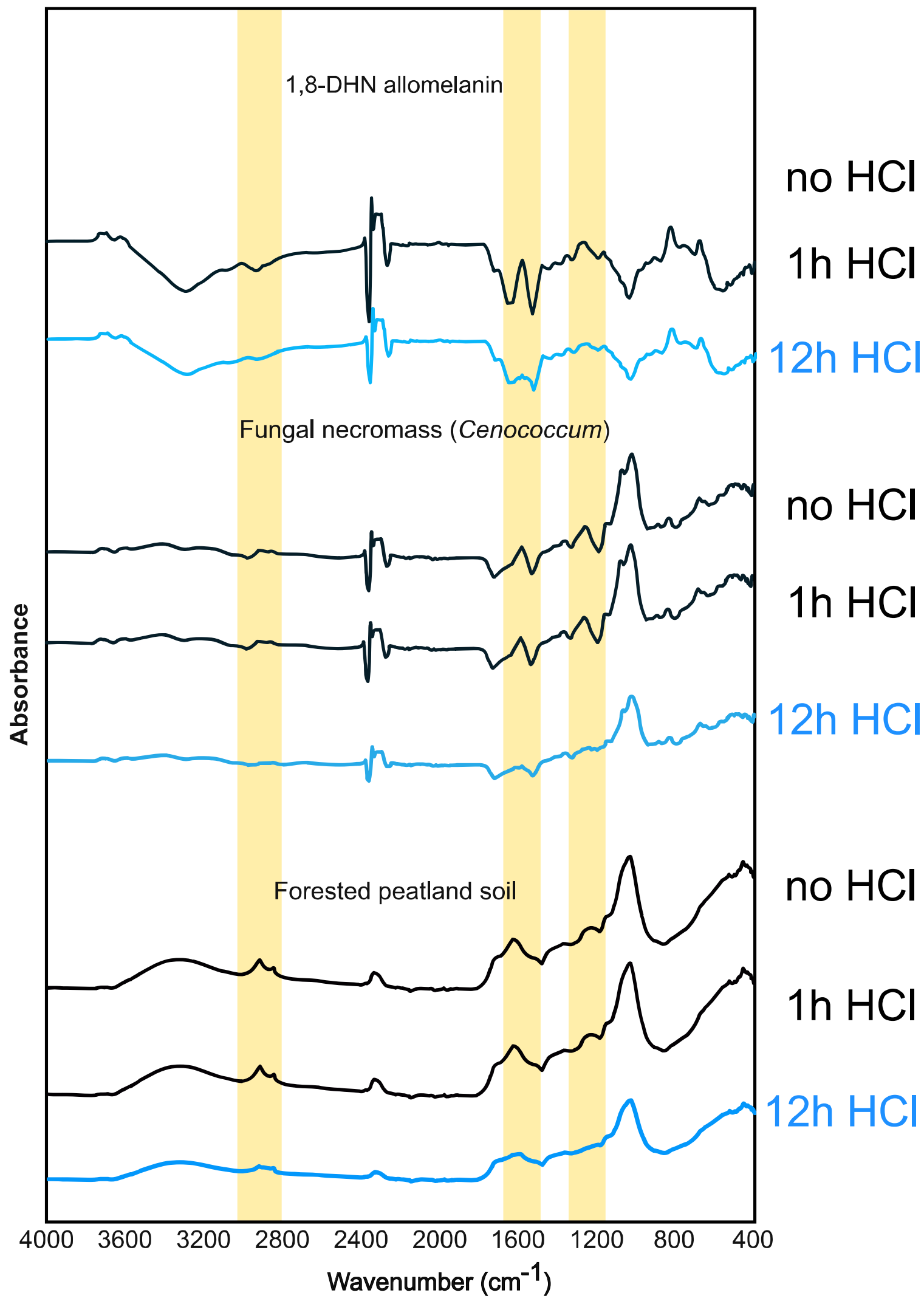

Supplementary Fig. 10. The effect of incubation with HCl (no HCl, 1h, 12h) on melanin structure. Marked in yellow column are the bands typical for melanin. After 12h HCl incubation there is a decrease in O-H/N-H band (3200-3400 cm^{-1}), C=C bands (1620-1510 cm^{-1}) and C-OH/C-N signals (1450-1200 cm^{-1}).

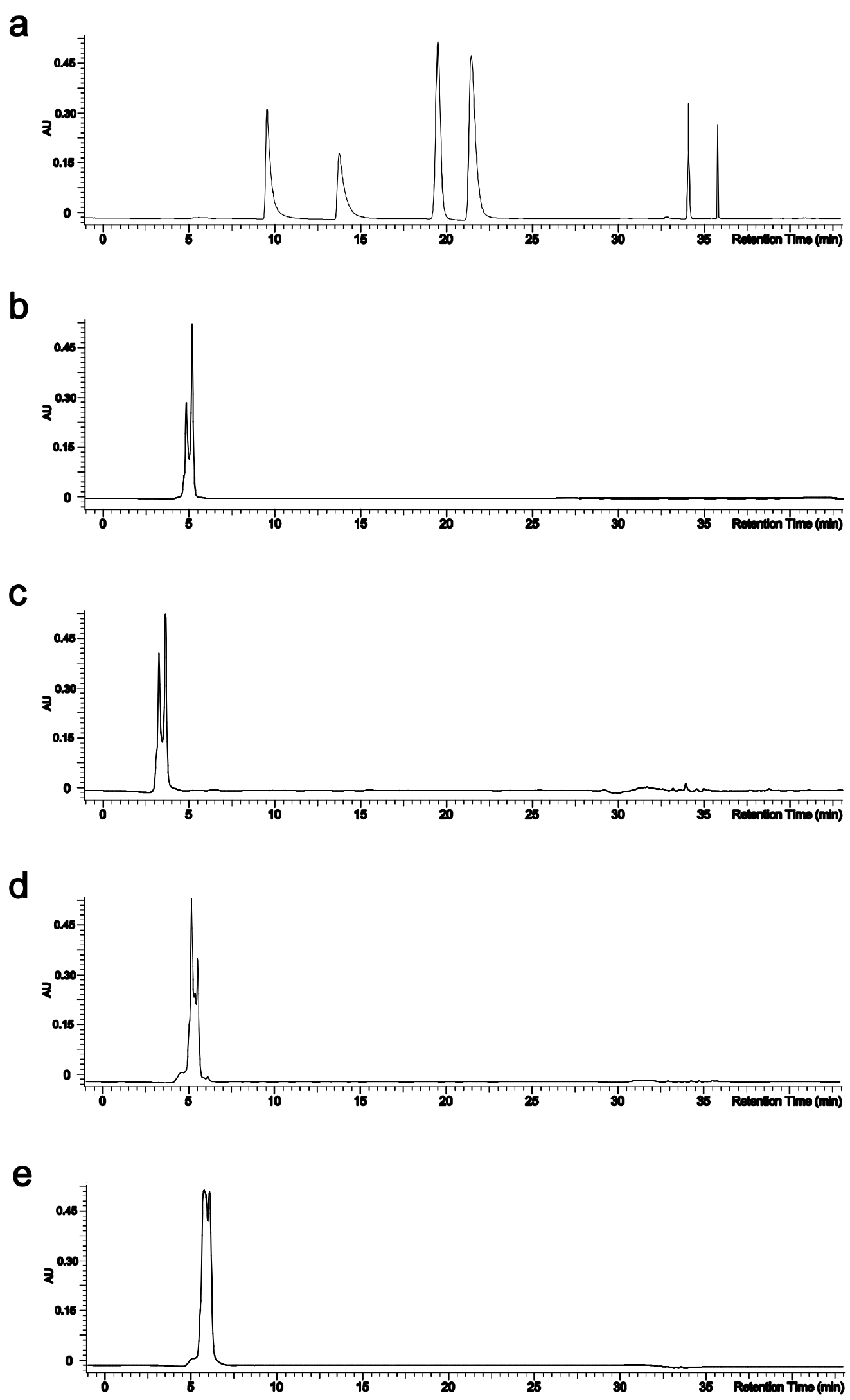

Supplementary Fig. 11. Chromatograms of products from the alkaline oxidation of non-melanin samples. a) standards all six markers for comparison purposes, b) pine seeds, c) pine roots, d) nut shells, e) condensed tannins extracted from spruce.

Supplementary Table 5. Ergosterol concentration in various non-melanin samples. Fungal biomass calculated with equation below[1].

Sample	Ergosterol µg g^{-1} DW	Fungal biomass mg g^{-1} DW
Pine seeds	-	-
Pine roots	2.0(0.03)	0.80(0.00)
Spruce seeds	-	-
Spruce roots	1.0(0.02)	0.40(0.00)
Pine cones	0.1(0.01)	0.04(0.00)
Spruce cones	0.1(0.02)	0.04(0.00)
Black oat grains	-	-
Nut shells	-	-
Lignin	-	-
Spruce tannins	-	-

$$FB(\mu g\ g-1\ DW) = ergosterol(\mu g\ g-1\ DW)\ x\ f\ x\ Rf$$

where f = 250 (1/4x1000, mg biomass µg^{-1} ergosterol) and Rf = 1.61 (correction factor for average recovery percent, 1/0.62).

Supplementary Table 6. Percent recovery of markers of melanin and melanin sources added to soil, average of three replicates.

soil	% recovery after addition to soil										
	TTCA	TDCA	PTCA	PDCA	PHA	IPHA	synthetic eumelanin	synthetic allomelanin	Sepia melanin	*Cenococcum*	*Tylospora*
Forest organic[A]	95	96	94	95	83	86	91	82	90	95	96
Forest mineral[A]	86	94	93	94	74	72	76	72	77	90	92
Forest organic[B]	93	93	92	95	85	87	92	80	90	93	95
Forest mineral[B]	86	89	90	87	77	72	83	73	81	88	89
Forest organic[C]	94	94	92	95	88	88	91	84	89	91	93
Forest mineral[C]	87	85	87	89	75	69	79	79	77	87	89
Forest organic[D]	96	94	90	92	83	87	86	85	84	94	95
Forest mineral[D]	85	89	88	88	81	72	72	79	71	89	90
Forested peatland[E]	94	97	96	97	87	84	90	86	88	92	94
Agricultural soil[F]	89	89	86	86	73	80	71	70	70	78	81
Agricultural soil[G]	80	85	86	87	71	69	67	69	68	77	80
Lufa soil[H]	88	93	92	96	88	80	84	84	85	85	87

Supplementary Table 7. Percent recovery of melanin added to clay minerals and iron oxides. In brackets standard deviation of three replicates.

**melanin/melanin marker/fungi**	**% recovery after interaction with soil minerals**			
	**illite**	**kaolinite**	**ferrihydrite**	**goethite**
TTCA	92(2.1)	88(2.4)	89(2.1)	94(0.6)
TDCA	95(2.1)	91(1.2)	92(1.4)	97(1.8)
PTCA	93(2.0)	89(1.8)	91(2.4)	94(1.6)
PDCA	97(1.0)	95(0.8)	96(1.5)	96(0.7)
PHA	95(1.1)	92(1.7)	94(1.3)	96(1.3)
IPHA	96(0.9)	93(1.7)	94(1.7)	95(0.5)
Sepia melanin	98(2.0)	96(1.0)	97(0.9)	96(2.4)
Synthetic eumelanin	97(3.1)	95(1.4)	96(0.8)	97(1.7)
Synthetic allomelanin	98(0.7)	94(0.8)	96(0.7)	97(1.8)
Cenococcum	99(0.3)	95(2.5)	97(0.8)	97(2.4)

Supplementary Table 8. Soil fungal necromass, total C and total N in soil samples. Mean value with standard deviation in brackets. DW – dry weight. Data for Forest soil[A] site taken from already published paper[9]. Statistically significant differences between soils are marked with different letters.

	**Fungal necromass mg g^{-1} DW**	**%C**	**%N**
Forest organic[A]	3.14(0.31)[ab]	26.5(1.95)[a]	0.61(0.07)[a]
Forest mineral[A]	0.02(0.00)[c]	2.56(0.40)[b]	0.08(0.00)[b]
Forest organic[B]	2.08(0.39)[b]	26.0(1.00)[a]	0.90(0.15)[ab]
Forest mineral[B]	0.19(0.04)[c]	2.59(0.30)[b]	0.09(0.01)[b]
Forest organic[C]	4.06(0.66)[a]	42.2(3.81)[a]	1.03(0.16)[a]
Forest mineral[C]	0.05(0.03)[c]	2.00(0.31)[bc]	0.12(0.06)[b]
Forest organic [D]	1.24(0.18)[b]	16.0(2.49)[a]	0.80(0.08)[a]
Forest mineral[D]	0.05(0.02)[c]	2.00(0.45)[bc]	0.12(0.04)[b]
Forested peatland[E]	2.25(0.42)[b]	50.9(1.30)[a]	1.56(0.21)[a]
Agricultural soil[F]	1.23(0.03)[b]	2.49(0.38)[b]	0.19(0.04)[b]
Agricultural soil[G]	0.92(0.21)[c]	1.40(0.09)[c]	0.18(0.01)[b]
Lufa standard soil[H]	1.00(0.19)[b]	2.04(0.36)[bc]	0.20(0.03)[b]

References

1. Wallander, H. *et al.* Evaluation of methods to estimate production, biomass and turnover of ectomycorrhizal mycelium in forests soils – A review. *Soil Biol. Biochem.* **57**, 1034–1047 (2013).
2. Adamczyk, B. *et al.* Nitrogen fertilisation of boreal forest soil increases soil carbon pool through elevated microbial necromass formation but also modifies tree secondary metabolism. *Soil Biol. Biochem.* **209**, 109917 (2025).
3. Adamczyk, B. *et al.* Plant roots increase both decomposition and stable organic matter formation in boreal forest soil. *Nat. Commun.* **10**, 3982 (2019).
4. Adamczyk, B., Kiikkilä, O., Kitunen, V. & Smolander, A. Can we measure condensed tannins from tannin–protein complexes? – A case study with acid–butanol assay in boreal forest soil organic layer. *Eur. J. Soil Biol.* **64**, 40–45 (2014).
5. Tikkasalo, O.-P. *et al.* Eddy-covariance fluxes of CO_2 , CH_4 and N_2 O in a drained peatland forest after clear-cutting. *Biogeosci.* **22**, 1277–1300 (2025).
6. Yli-Halla, M. & Mokma, D. L. Soils in an agricultural landscape of Jokioinen, south-western Finland. *AFSci* **10**, 33–43 (2001).
7. Šmídová, K. *et al.* Conventional and biodegradable agricultural microplastics: effects on soil properties and microbial functions across a European pedoclimatic gradient. *Environ. Pol.* **386**, 127212 (2025).
8. Adamczyk, S. *et al.* Biodegradable microplastics induce profound changes in lettuce (Lactuca sativa) defense mechanisms and to some extent deteriorate growth traits. *Environ. Pol.* **363**, 125307 (2024).
9. Adamczyk, B. *et al.* Nitrogen fertilisation of boreal forest soil increases soil carbon pool through elevated microbial necromass formation but also modifies tree secondary metabolism. *Soil Biol. Biochem.* **209**, 109917 (2025).

10. Ito, S. & Wakamatsu, K. Chemical degradation of melanins: application to identification of dopamine-melanin. *Pigment Cell Res.* **11**, 120–126 (1998).

11. Nezirević, D., Årstrand, K. & Kågedal, B. Hydrophilic interaction liquid chromatographic analysis of aminohydroxyphenylalanines from melanin pigments. *J. Chromatogr. A* **1163**, 70–79 (2007).
