# Supplementary material for: A method quantifying soil melanin to understand fungal impacts on soil carbon stability
Source: Nat Commun. 2026 Oct 11;17:11524. doi: 10.1038/s41467-026-78616-4 (PMC13745510; doi:10.1038/s41467-026-78616-4)
Supplement: Supplementary file 3 [file 41467_2026_78616_MOESM3_ESM.pdf]

nature portfolio

Peer Review File

A method quantifying soil melanin to understand fungal impacts on soil carbon stability.

Corresponding Author: Dr Bartosz Adamczyk

This file contains all reviewer reports in order by version, followed by all author rebuttals in order by version.

Version 0:

Reviewer comments:

Reviewer #1

(Remarks to the Author)
This work establishes a method for analyzing melanin produced by fungi in soil. While the development of a new analytical method is valuable and worth sharing with the scientific community, I do not believe this paper meets the impact or readership scope expected for Nature Communications.

The authors have not clearly articulated the importance of this work. Beyond fungal sources, how much melanin in soil is expected to originate from other organisms or sources? Why is it important to specifically analyze fungal-derived melanin? What are the potential application scenarios for this method? Furthermore, what significant advancements or methodological improvements does this approach offer compared with existing techniques? Finally, what novel insights does this method provide, or is expected to provide, about melanin in soil ecosystems?

In addition, the title is overly general and somewhat misleading, and should be revised to more accurately reflect the content and scope of the study.

Reviewer #2

(Remarks to the Author)
1. The overall innovation is insufficient, and the workload is minimal. It merely proposes a method for detecting fungal melanin in soil, which is actually a rather weak innovation point. The methods employed are essentially mainstream and already established, such as the detection of eumelanins and pheomelanins markers, with related HPLC and HPLC-MS detection techniques having been reported previously.
2. The only novel aspect is the proposal of allomelanins detection markers, but this section is merely a brief sub-topic under the broader theme of soil fungal melanin detection in this paper. It is not emphasized as a key point and lacks sufficient depth or complexity in its discussion.
3. This paper emphasizes the detection of fungal melanin in soil, but the detection method measures the content of all melanin oxidation markers present in the soil. However, soil may also contain plant-derived melanin, such as certain fallen seeds and the degradation products of plants after melanin-containing plants die. This plant-derived melanin is included in the detection process, making it impossible to accurately and effectively reflect the content of fungal melanin. Moreover, the soil sample processing method is unclear, and the soil samples are not representative.
4. Recommendation: The experiments related to allomelanins markers can be further improved and enriched by testing various biological samples containing allomelanins.
5. Inconsistent reference formats, such as journal name abbreviations, capitalization of paper titles, and other issues.

Reviewer #3

(Remarks to the Author)
This manuscript addresses a significant methodological gap in soil microbial ecology — the direct quantification and differentiation of fungal melanin types in soil matrices. The proposed HPLC-based approach appears robust and holds strong potential for studies on soil carbon stabilization.
However, several aspects of the paper need clarification, refinement, and additional validation details before it can be

considered for publication.
Overall, the study presents valuable scientific insights, but logical flow and methodological explanation are recommended to enhance its clarity, reproducibility, and overall impact.
Abstract:
1. Adding a statement to explicitly state how this method differs from existing Azure A–based or DNA-based techniques would clarify novelty.
2. The second sentence (“Here, we propose a method...”) could be combined with the last one to create a more refined flow.
3. Avoid overly general terms like “precisely measure”. Instead, use “quantitatively determine” or “accurately quantify.”
Introduction:
4. The heading for Introduction section is missing.

5. Start the introduction with soil organic matter and its stabilization, then move to role of fungi and melanin, and finally to methodological limitations for a more logical flow. The classification paragraph could be shortened since it interrupts the narrative.

6. End the introduction by clearly stating: “Therefore, this study aims to develop and validate a liquid chromatography method for direct quantification and differentiation of fungal melanins in soil.” to clarify the gap and the research question.

7. Replace “melanin content is dependent…” with “melanin production varies with environmental conditions.”

Results and Discussion:
8. Compare performance (such as sensitivity, reproducibility) with existing melanin detection techniques (e.g., Azure A spectrophotometry, FTIR).

9. Consider separating results and discussion sections that are currently merged. Quantitative results (data, figures) and interpretative discussion can be separated for better clarity.

10. Include error bars in data tables or graphs, if possible for statistical robustness.

11. Discuss how measured melanin concentrations vary between soil types and what that implies for carbon persistence for ecological relevance.

Methods:
12. Clarify whether blank soil samples (melanin-free) or non-fungal organic matter controls were analyzed to confirm specificity.

13. Suggestion to add a validation section and include intra- and inter-day precision, repeatability, and recovery test results.

14. Provide explicit equations for converting oxidation product concentrations to melanin equivalents (using the mentioned factors 200 and 333).

15. Correct typo errors like “isophthtalic” to “isophthalic.” And ensure consistent units (mg mL^{-1} or mg/mL) throughout.

Conclusions:
16. Replace “creates a basis for addressing fungal melanin's function in global C cycle” with “provides a foundational tool for investigating fungal melanin’s contribution to soil carbon persistence.”
17. Ensure consistent use of abbreviations (e.g., HPLC, UV, TFA, MS — define at first mention).

Version 1:

Reviewer comments:

Reviewer #1

(Remarks to the Author)
The authors addressed my comments mostly.

Reviewer #2

(Remarks to the Author)
1.This paper actually investigates a soil digestion method that enables the detection of melanin by high-performance liquid chromatography. It demonstrates a certain degree of novelty but lacks significant conceptual breakthroughs.
2. This paper derives a formula for calculating the amount of natural melanin based on the marker production from three types of synthetic melanin under alkaline oxidation, which lacks strong scientific validity. Even for eumelanin, the generation of its two oxidative products depends on the composition and ratio of its monomers (DHI and DHICA). Currently, the proportion of eumelanin monomers is not a fixed value and varies among species. Relying solely on the proposed calculation formula for synthetic melanin is unreliable.
3. The biological environment of the soil is relatively complex. The method described in this paper primarily aims to provide

a more accurate estimation of soil fungal melanin compared to previous methods. Since the sources of eumelanin are extremely diverse, the previously mentioned potential melanin-containing plants only represent one aspect of possible interference, while animal-derived melanin is actually the most critical issue. Numerous animal species contain eumelanin, and after the decomposition of animal remains, melanin may accumulate in the soil, causing significant interference. Moreover, the extent of this interference is highly difficult to quantify, and the degree of interference varies among soils from different environments.

Reviewer #3

(Remarks to the Author)
This study presents a new high-performance liquid chromatography (HPLC) method for accurately quantifying and classifying melanins in soil. Melanins contained in fungal necromass play an important role in long-term carbon storage, yet conventional spectroscopic and DNA analysis lack precision and specificity. The authors established a method using alkaline hydrogen oxidation to distinguish three fungal-specific melanins (eumelanin, pheomelanin, and allomelanin). Verification using this method demonstrated high measurement accuracy, eliminating interference from plant components. Furthermore, soil melanin concentrations were found to be strongly correlated with carbon content, contributing to improved carbon cycle models. However, to improve the experimental reliability of this paper, the following revisions are proposed:

1. Reselection of Standards and Verification of Coefficients
(a) Regarding the validity of the "conversion coefficients" that form the quantitative basis of this paper, we recommend changing the eumelanin standard materials. The synthetic eumelanin (M8631) manufactured by Sigma-Aldrich used in this study may have different chemical properties, such as being soluble in weakly alkaline aqueous solutions, unlike natural eumelanin (which is sparingly soluble) (d'Ischia et al., Pigment Cell Melanoma Rers. 2013, 26, 616-633). Sepia melanin (derived from squid ink), which has a structure closer to natural eumelanin, should be used as the standard substance (M2649) to reexamine the appropriateness of the current "200-fold" factor. Adopting sepia melanin as the standard substance would eliminate the uncertainty associated with structural differences between synthetic and natural products and improve the absolute reliability of soil melanin amounts calculated using this method. In particular, the "200-fold" factor proposed in this paper would be revised to a value more closely based on the "natural standard."
(b) Consideration of the universality of the coefficients: The coefficients "333-fold" for allomelanin and "200-fold" for eumelanin are based on the yields of specific synthetic products. Further discussion is needed regarding whether these fixed coefficients are applicable to "natural soil melanins" that have been altered or degraded in the environment, or whether adjustments based on the degree of polymerization and oxidation are necessary. The coefficient "333" for the synthetic allomelanin used in this study may also need to be verified and revised. The authors synthesized 1,8-DHN by exposing it to air in a 0.01 M NaOH aqueous solution for 48 hours (autoxidation). This autoxidation synthesis may differ in the degree of polymerization and structural complexity from the actual "enzymatic synthesis" by fungi. If natural allomelanin had a more rigid structure (resistance to degradation), the yield of the oxidation marker would be lower than 3 mg and the coefficient would be even larger than 333. The paper states that fungal melanin is incorporated into the cell wall. However, the standard material used in the experiment was a pure precipitate. Natural soil melanin may be complex intertwined with other cell wall components (proteins and polysaccharides), which may affect the efficiency of alkaline oxidation. This reaction resulted in a very low yield (0.3%), ranging from 1 g to 3 mg. Therefore, slight differences in the polymerization state of allomelanin could lead to significant fluctuations in the yield, resulting in a large multiplier of 333.

2. Evaluation of Pheomelanin as an Analytical Marker:
In this paper, TTCA and TDCA are used as chemical degradation markers for the benzothiazole unit of pheomelanin. Measurement of 4-AHP (a reductive hydrolysis product by HI), a marker for benzothiazine, another major building block of pheomelanin, should also be considered to improve the comprehensiveness of the method (Wakamatsu et al., Pigment Cell Res., 2002, 15, 225-232), or a detailed chemical justification for the sufficiency of oxidation indicators alone should be provided.
Note that ref. 19 (Ito, S. & Jimbow, K., J. Invest. Dermatol., 1983) is an outdated chemical analysis method and should be replaced with Ito, S., et al. Pigment Cell Melanoma Res., 2011, 24, 605-613.

3. Optimization of pretreatment protocol and verification of robustness:
We seek suggestions for modifications to address chemical and physical interferences in the following extraction processes.

a) Evaluation of the impact of acid treatment (6 M HCl): The impact of hydrochloric acid treatment at 80°C for 1 hour, performed to remove impurities, on the structure of melanin itself and the efficiency of marker production by subsequent alkaline oxidation should be thoroughly investigated.
b) Influence of soil matrix (minerals): Although recovery rates (f) have been calculated for some soil types (approximately 80% for allomelanin), additional validation data under a wider variety of soil conditions is needed to clarify the impact of strong adsorption to clay minerals and iron oxides on extraction efficiency.

4. Improving comparative accuracy as an ecological indicator:
We request the following revisions regarding the comparison targets to assert melanin's superiority.
This paper suggests that melanin may be a better indicator for predicting soil carbon stability than amino sugars (necromass indicators). However, because amino sugars also contain bacterial components, future research should strengthen comparisons with ergosterol (a viable cell indicator) and other biomarkers more specific to fungi to more clearly distinguish melanin's uniqueness and long-term preservation potential.

Version 2:

Reviewer comments:

Reviewer #4

(Remarks to the Author)
The research team has provided extremely honest and scientifically valid responses to Reviewer 4's concerns, significantly improving the paper's credibility.
In particular, core issues such as the validity of the standard material and the accuracy of the coefficients, which had been a concern in previous discussions, have been improved through concrete additional experiments.
In conclusion, the authors have not only revised the text but have also completely addressed the reviewer's concerns by adding empirical data such as recalculations using sepia melanin, comparative experiments with 4-AHP, and mineral adsorption experiments. This response makes the HPLC method proposed in this paper a more robust method for quantifying soil melanin.

Open Access This Peer Review File is licensed under a Creative Commons Attribution 4.0 International License, which permits use, sharing, adaptation, distribution and reproduction in any medium or format, as long as you give appropriate credit to the original author(s) and the source, provide a link to the Creative Commons license, and indicate if changes were made.
In cases where reviewers are anonymous, credit should be given to 'Anonymous Referee' and the source.
The images or other third party material in this Peer Review File are included in the article’s Creative Commons license, unless indicated otherwise in a credit line to the material. If material is not included in the article’s Creative Commons license and your intended use is not permitted by statutory regulation or exceeds the permitted use, you will need to obtain permission directly from the copyright holder.
To view a copy of this license, visit https://creativecommons.org/licenses/by/4.0/

REVIEWER COMMENTS

Reviewer #1 (Remarks to the Author):

<u>This work establishes a method for analyzing melanin produced by fungi in soil. While the development of a new analytical method is valuable and worth sharing with the scientific community, I do not believe this paper meets the impact or readership scope expected for Nature Communications. The authors have not clearly articulated the importance of this work. Beyond fungal sources, how much melanin in soil is expected to originate from other organisms or sources?</u>

Thank you for this suggestion. We revised the abstract and introduction to more clearly articulate the broader significance of this work, particularly its relevance for elucidating mechanisms of long-term soil carbon stabilization derived from fungal necromass (*lines 13–22; 27–49, in MS version with marked changes*). In addition, we expanded the manuscript to address non-fungal sources of melanin-like compounds, demonstrating that while plant phenolics can form dark polymers, only fungi produce 1,8-DHN allomelanin, and our method is specific to this fungal melanin type (lines 103–112; 239–247). By incorporating additional reference materials and controls, we now demonstrate that our analytical approach is selective for fungal 1,8-DHN allomelanin and does not respond to plant-derived phenolic polymers. This specificity allows, for the first time, the unambiguous detection and quantification of fungal melanin in complex soil matrices. We therefore believe that the method provides a critical advance for soil biogeochemistry by enabling direct investigation of fungal contributions to stable soil carbon pools, a question of broad relevance to carbon cycling and climate-related research.

<u>Why is it important to specifically analyze fungal-derived melanin? What are the potential application scenarios for this method?</u>

We clarified and underlined the need to understand and quantify soil melanin in both abstract and in Introduction (lines 13-22; 27-49). In short, fungal melanin is supposed to be crucial in C and nutrient cycling (including C stabilization) across terrestrial ecosystems (see e.g. Netherway and Bahram, 2024, Trends in Microbiology). The method presented here enables targeted detection of fungal melanin in soils, providing new opportunities to investigate fungal contributions to stable soil organic matter, assess carbon persistence across ecosystems, and improve biogeochemical models that explicitly incorporate microbial-derived carbon.

<u>Furthermore, what significant advancements or methodological improvements does this approach offer compared with existing techniques?</u>

Thank you for raising this point. Existing analytical techniques cannot reliably quantify melanin in soils or discriminate among melanin types under complex soil conditions. We demonstrate that commonly used methods such as Azure A spectrophotometry and FTIR lack melanin specificity in soils due to strong overlap with lignin, tannins, and other polyphenolic compounds, rendering them unsuitable for soil samples despite their applicability to pure fungal biomass (lines 114–131; 248–279).

<u>Finally, what novel insights does this method provide, or is expected to provide, about melanin in soil ecosystems?</u>

We clarified the meaning of precise melanin measurements in developed introduction and discussion. Melanin is expected to play crucial role in C and nutrient cycling, and especially in C stabilization in the soil, one of the most urgent topics in ecology and land management (see Netherway and Bahram, 2024, Trends in Microbiology) (lines 13-22; 27-49, 299-304). On the contrary, studies on melanin in the field of medicine and technology are far more developed, for example by pioneers in the field of melanin, professors Kawamatsu and Ito (https://doi.org/10.1034/j.1600-0749.13.s8.19.x and other papers cited in revised version of our MS) (lines 59-62; 65-69).

<u>In addition, the title is overly general and somewhat misleading and should be revised to more accurately reflect the content and scope of the study.</u>

We modified the title in line with this comment: “A method quantifying soil melanin to understand fungal impacts on soil carbon stability”

Reviewer #2 (Remarks to the Author):

1. The overall innovation is insufficient, and the workload is minimal. It merely proposes a method for detecting fungal melanin in soil, which is actually a rather weak innovation point. The methods employed are essentially mainstream and already established, such as the detection of eumelanins and pheomelanins markers, with related HPLC and HPLC-MS detection techniques having been reported previously.

Thank you for this valuable feedback, which allowed us to strengthen the Introduction and clarify the need for our method. At present, no method exists for measuring melanin in soil samples, despite the clear need to elucidate the role of melanized fungal necromass in carbon stabilization (see Netherway & Bahram, 2024, *Trends in Microbiology*). Although eumelanin and pheomelanin have previously been detected using HPLC, these studies were limited to non-soil materials (e.g., heather) and have not been applied to soil matrices. Notably, even recent ground-breaking work by Professors Kawamatsu and Ito (10.3390/ijms24098305) does not provide a method suitable for soil samples. We have also clarified in both the Introduction and the Abstract why the development of a soil-specific melanin quantification method is needed (lines 13-22; 27-49; *in MS version with marked changes*).

2. The only novel aspect is the proposal of allomelanins detection markers, but this section is merely a brief sub-topic under the broader theme of soil fungal melanin detection in this paper. It is not emphasized as a key point and lacks sufficient depth or complexity in its discussion.

We have revised the Abstract and Introduction to more clearly emphasize both the novelty of our approach and the need for such a method (see also responses above) (lines lines 13-22; 27-49, 299-304). In the Discussion, we have placed greater focus on allomelanin and separated its interpretation from the Results section for improved clarity. To our knowledge, none of the melanin types examined here—pheomelanin, eumelanin, or allomelanin—have previously been studied in soil.

3. This paper emphasizes the detection of fungal melanin in soil, but the detection method measures the content of all melanin oxidation markers present in the soil. However, soil may also contain plant-derived melanin, such as certain fallen seeds and the degradation products of plants after melanin-containing plants die. This plant-derived melanin is included in the detection process, making it impossible to accurately and effectively reflect the content of fungal melanin.

We tested plant-derived melanin-like compounds and confirmed that our method does not detect them. Unlike fungi, plants cannot produce allomelanin via the 1,8-DHN pathway; their melanin-like compounds are oxidized and polymerized polyphenols. We have clarified this distinction in the Discussion and included plant-derived materials specifically to verify this point (lines 222-247). In addition, soil samples are routinely sieved through a 2 mm mesh (as in our protocol), which removes larger plant litter fragments (lines 373-380).

Moreover, the soil sample processing method is unclear, and the soil samples are not representative.

We clarified the steps of the method, added a diagram to clearly illustrate its workflow, and included additional soil samples (and non-soil materials) in the study (lines lines 373-380, Fig. 1, lines 103-112). The aim (as introduced in introduction) is to study melanin in the soil (lines 13-22; 27-49, 299-304).

4. Recommendation: The experiments related to allomelanins markers can be further improved and enriched by testing various biological samples containing allomelanins.

We validated our method with a wide set of plant derived materials and compounds. We proved that our method is specific for 1,8-DHN allomelanin (lines 103-112; 239-247).

5. Inconsistent reference formats, such as journal name abbreviations, capitalization of paper titles, and other issues.

References fixed.

Reviewer #3 (Remarks to the Author):

This manuscript addresses a significant methodological gap in soil microbial ecology — the direct quantification and differentiation of fungal melanin types in soil matrices. The proposed HPLC-based approach appears robust and holds strong potential for studies on soil carbon stabilization. However, several aspects of the paper need clarification, refinement, and additional validation details before it can be considered for publication. Overall, the study presents valuable scientific insights, but logical flow and methodological explanation are recommended to enhance its clarity, reproducibility, and overall impact.

Thank you for these constructive comments. We have carefully followed your suggestions to improve the manuscript, enhancing clarity, logical flow, and methodological detail to support reproducibility and highlight the significance of our approach.

Abstract:

1. Adding a statement to explicitly state how this method differs from existing Azure A–based or DNA-based techniques would clarify novelty.
2. The second sentence ("Here, we propose a method...") could be combined with the last one to create a more refined flow.
3. Avoid overly general terms like "precisely measure". Instead, use "quantitatively determine" or "accurately quantify."

Answer: the abstract is rewritten in line with these suggestions (lines 14-24, *in MS version with marked changes*)

Introduction:

4. The heading for Introduction section is missing.
5. Start the introduction with soil organic matter and its stabilization, then move to role of fungi and melanin, and finally to methodological limitations for a more logical flow. The classification paragraph could be shortened since it interrupts the narrative.
6. End the introduction by clearly stating: "Therefore, this study aims to develop and validate a liquid chromatography method for direct quantification and differentiation of fungal melanins in soil." to clarify the gap and the research question.
7. Replace "melanin content is dependent…" with "melanin production varies with environmental conditions."

Answer: All done as proposed (lines 26, 26-49; 65, 52)

Results and Discussion:

8. Compare performance (such as sensitivity, reproducibility) with existing melanin detection techniques (e.g., Azure A spectrophotometry, FTIR).

We applied both the Azure A method and FTIR analyses to soil samples, fungal samples, plant-derived materials, and synthetic melanins. For both approaches, the spectral features or signals associated with melanin substantially overlap with those of polyphenolic compounds, resulting in no specificity. Consequently, neither method is well suited for complex soil matrices. Instead, these techniques are more appropriate to lab cultivated fungal necromass, where the absence of lignin and tannins minimizes spectral overlap and improves interpretability (lines 114-120; 248-279).

9. Consider separating results and discussion sections that are currently merged. Quantitative results (data, figures) and interpretative discussion can be separated for better clarity.

We followed this suggestion, now Results and Discussion are separated, Results section starting from line 83, Discussion from line 212).

10. Include error bars in data tables or graphs, if possible for statistical robustness.

Done

11. Discuss how measured melanin concentrations vary between soil types and what that implies for carbon persistence for ecological relevance.

We added such analyzes and paragraph to discussion (lines 281-304).

Methods:

12. Clarify whether blank soil samples (melanin-free) or non-fungal organic matter controls were analyzed to confirm specificity.

Yes, we analyzed a wide range of melanin-free materials to assess the specificity of our protocol. These included plant-derived materials (e.g., seeds and cones) as well as pure chemical compounds (lignin and condensed tannins). The same set of additional samples was also used to evaluate the specificity of the Azure A and FTIR methods toward melanin. These results are presented in the Results section and discussed in depth in the Discussion. (lines 103-112, 239-247, 444-451)

13. Suggestion to add a validation section and include intra- and inter-day precision, repeatability, and recovery test results.

14. Provide explicit equations for converting oxidation product concentrations to melanin equivalents (using the mentioned factors 200 and 333).

15. Correct typo errors like “isophthtalic” to “isophthalic.” And ensure consistent units (mg mL^{-1} or mg/mL) throughout.

All done as suggested (lines 148-160; 174-181; 314; 678).

Conclusions:

16. Replace “creates a basis for addressing fungal melanin's function in global C cycle” with “provides a foundational tool for investigating fungal melanin’s contribution to soil carbon persistence.”

Done (lines 302-304).

17. Ensure consistent use of abbreviations (e.g., HPLC, UV, TFA, MS — define at first mention).

Done.

REVIEWER COMMENTS

Reviewer #1 (Remarks to the Author):

The authors addressed my comments mostly.

Thank you!

Reviewer #2 (Remarks to the Author):

1. This paper actually investigates a soil digestion method that enables the detection of melanin by high-performance liquid chromatography. It demonstrates a certain degree of novelty but lacks significant conceptual breakthroughs.

Thank you for this comment! It helped us to restructure the MS from merely method development MS into more conceptual MS. We modified and added a couple of sentences to the MS to underline conceptual breakthroughs, lines 20, 65, 70, 76, 80, 253, 354, 389, 393. (line numbering in clean version of MS, not with marked changes).

2. This paper derives a formula for calculating the amount of natural melanin based on the marker production from three types of synthetic melanin under alkaline oxidation, which lacks strong scientific validity. Even for eumelanin, the generation of its two oxidative products depends on the composition and ratio of its monomers (DHI and DHICA). Currently, the proportion of eumelanin monomers is not a fixed value and varies among species. Relying solely on the proposed calculation formula for synthetic melanin is unreliable.

In line with this comment and Reviewer 4 we used sepia melanin instead of synthetic melanin for calculations, and instead of synthetic allomelanin we used as standard allomelanin from *Cenococcum* fungi and we modified our formulas, line 201.

3. The biological environment of the soil is relatively complex. The method described in this paper primarily aims to provide a more accurate estimation of soil fungal melanin compared to previous methods.

There is no previous method to estimate directly soil melanin. Studies of melanin in different matrices (skin, etc.) are very well developed (e.g. the group of Professors Wakamatsu and Ito), but current methods for melanin are used for non-soil samples. DNA-based methods are only estimating genetic potential to produce melanin. We clarified all these issues in lines 14-22, 52,58, 65, 389.

Since the sources of eumelanin are extremely diverse, the previously mentioned potential melanin-containing plants only represent one aspect of possible interference, while animal-derived melanin is actually the most critical issue. Numerous animal species contain eumelanin, and after the decomposition of animal remains, melanin may accumulate in the soil, causing significant interference. Moreover, the extent of this interference is highly difficult to quantify, and the degree of interference varies among soils from different environments.

Thank you for this challenging comment. We discussed interference of melanin concentrations by animal sources in line 356.

Reviewer #4 (Remarks to the Author):

This study presents a new high-performance liquid chromatography (HPLC) method for accurately quantifying and classifying melanins in soil. Melanins contained in fungal necromass play an important role in long-term carbon storage, yet conventional spectroscopic and DNA analysis lack precision and specificity. The authors established a method using alkaline hydrogen oxidation to distinguish three

fungal-specific melanins (eumelanin, pheomelanin, and allomelanin). Verification using this method demonstrated high measurement accuracy, eliminating interference from plant components. Furthermore, soil melanin concentrations were found to be strongly correlated with carbon content, contributing to improved carbon cycle models.
Thank you!

However, to improve the experimental reliability of this paper, the following revisions are proposed:
1. Reselection of Standards and Verification of Coefficients
(a) Regarding the validity of the "conversion coefficients" that form the quantitative basis of this paper, we recommend changing the eumelanin standard materials. The synthetic eumelanin (M8631) manufactured by Sigma-Aldrich used in this study may have different chemical properties, such as being soluble in weakly alkaline aqueous solutions, unlike natural eumelanin (which is sparingly soluble) (d'Ischia et al., Pigment Cell Melanoma Rers. 2013, 26, 616-633). Sepia melanin (derived from squid ink), which has a structure closer to natural eumelanin, should be used as the standard substance (M2649) to reexamine the appropriateness of the current "200-fold" factor. Adopting sepia melanin as the standard substance would eliminate the uncertainty associated with structural differences between synthetic and natural products and improve the absolute reliability of soil melanin amounts calculated using this method. In particular, the "200-fold" factor proposed in this paper would be revised to a value more closely based on the "natural standard."
Thank you for these very valuable comments! Now for eumelanin we used Sepia melanin instead of synthetic eumelanin, and we modified the formulas accordingly avoiding “generalized” factors, see line 201 (line numbering in clean version of MS, not with marked changes). We also adjusted calculations and statistics and figures in line with new formulas.

(b) Consideration of the universality of the coefficients: The coefficients "333-fold" for allomelanin and "200-fold" for eumelanin are based on the yields of specific synthetic products. Further discussion is needed regarding whether these fixed coefficients are applicable to "natural soil melanins" that have been altered or degraded in the environment, or whether adjustments based on the degree of polymerization and oxidation are necessary. The coefficient "333" for the synthetic allomelanin used in this study may also need to be verified and revised. The authors synthesized 1,8-DHN by exposing it to air in a 0.01 M NaOH aqueous solution for 48 hours (autoxidation). This autoxidation synthesis may differ in the degree of polymerization and structural complexity from the actual "enzymatic synthesis" by fungi. If natural allomelanin had a more rigid structure (resistance to degradation), the yield of the oxidation marker would be lower than 3 mg and the coefficient would be even larger than 333. The paper states that fungal melanin is incorporated into the cell wall. However, the standard material used in the experiment was a pure precipitate. Natural soil melanin may be complex intertwined with other cell wall components (proteins and polysaccharides), which may affect the efficiency of alkaline oxidation. This reaction resulted in a very low yield (0.3%), ranging from 1 g to 3 mg. Therefore, slight differences in the polymerization state of allomelanin could lead to significant fluctuations in the yield, resulting in a large multiplier of 333.
In line with this comment, for allomelanin we used *Cenococcum* fungi which is allomelanin-rich. We modified also formulas, avoiding “universal” coefficients, lines

201. We also adjusted calculations and statistics and figures in line with new formulas.

2. Evaluation of Pheomelanin as an Analytical Marker:
In this paper, TTCA and TDCA are used as chemical degradation markers for the benzothiazole unit of pheomelanin. Measurement of 4-AHP (a reductive hydrolysis product by HI), a marker for benzothiazine, another major building block of pheomelanin, should also be considered to improve the comprehensiveness of the method (Wakamatsu et al., Pigment Cell Res., 2002), or a detailed chemical justification for the sufficiency of oxidation indicators alone should be provided.
We performed additional analyses using 4-AHP and 3-AHP (AT) markers following reductive hydrolysis, as recommended. The results obtained were added to the revised manuscript (lines 108 and 325 in the clean version). We also compared these results with those obtained using our proposed alkaline oxidation method. Under alkaline oxidation conditions, pheomelanin markers (TTCA and TDCA) were detectable in chromatograms. In contrast, reductive hydrolysis of soil samples did not produce chromatograms of sufficient quality for reliable quantitative analysis. The low abundance of pheomelanin markers in soil samples is chemically and biologically reasonable, as pheomelanin is rare in fungi, while animal-derived pheomelanin contributions to soil melanin are expected to be minor (see discussion in line 356). Therefore, alkaline oxidation-based indicators were considered sufficient for characterization of pheomelanin in the investigated soil matrices.

Note that ref. 19 (Ito, S. & Jimbow, K., J. Invest. Dermatol., 1983) is an outdated chemical analysis method and should be replaced with Ito, S., et al. Pigment Cell Melanoma Res., 2011, 24, 605-613.
Done as suggested, line 70.

3. Optimization of pretreatment protocol and verification of robustness:
We seek suggestions for modifications to address chemical and physical interferences in the following extraction processes.
a) Evaluation of the impact of acid treatment (6 M HCl): The impact of hydrochloric acid treatment at 80°C for 1 hour, performed to remove impurities, on the structure of melanin itself and the efficiency of marker production by subsequent alkaline oxidation should be thoroughly investigated.
We added analyzes of the effect of HCl incubation time and temperature on further alkaline oxidation (Supplementary Table 3, 4), we also analyzed changes in melanin structure with FTIR, line 117 (Supplementary Fig. 10).

b) Influence of soil matrix (minerals): Although recovery rates (f) have been calculated for some soil types (approximately 80% for allomelanin), additional validation data under a wider variety of soil conditions is needed to clarify the impact of strong adsorption to clay minerals and iron oxides on extraction efficiency.
% recovery was tested for every used soil samples (see Table 2, where in caption it is written “averaged for all used soils”; we also provided all data for separate soils in Supplementary Table 9. Moreover, we added % recovery of melanin markers and melanin after their interaction with soil minerals (kaolinite, illite, ferrihydrite, goethite, see Supplementary Table 7), and we discussed it in Discussion, line 380.

4. Improving comparative accuracy as an ecological indicator:
We request the following revisions regarding the comparison targets to assert

melanin's superiority. This paper suggests that melanin may be a better indicator for predicting soil carbon stability than amino sugars (necromass indicators). However, because amino sugars also contain bacterial components, future research should strengthen comparisons with ergosterol (a viable cell indicator) and other biomarkers more specific to fungi to more clearly distinguish melanin's uniqueness and long-term preservation potential.

Thank you for raising this issue. Amino sugars include muramic acid (marker of bacterial necromass) and glucosamine (marker of fungal necromass). Fungal necromass is always calculated by subtracting bacterial glucosamine (GlcN) from total GlcN and based on the average molar ratio of MurA and GlcN in bacterial cells of 1–2 (Amelung et al. 1999, Adamczyk et al. 2024). Thus, our results of fungal necromass are not overestimated via bacterial necromass. Moreover, ergosterol is a direct marker of fungi (Adamczyk et al 2024, New Phytologist), however, it is only a marker of fungal biomass, not necromass. Living fungal biomass is a great decomposer of organic matter, thus it cannot be seen as element of stable C marker. We modified the sentence in our MS, line 346 to underline that melanin may be an important addition to fungal necromass (amino sugars) in disentangling the role of fungal necromass in soil C stabilization.
